# Screening for Innovative Sources of Carotenoids and Phenolic Antioxidants among Flowers

**DOI:** 10.3390/foods10112625

**Published:** 2021-10-29

**Authors:** Antonio J. Meléndez-Martínez, Ana Benítez, Mireia Corell, Dolores Hernanz, Paula Mapelli-Brahm, Carla Stinco, Elena Coyago-Cruz

**Affiliations:** 1 Food Colour and Quality Laboratory, Facultad de Farmacia, Universidad de Sevilla, 41012 Sevilla, Spain; ajmelendez@us.es (A.J.M.-M.); abenitez@us.es (A.B.); pmapelli@us.es (P.M.-B.); cstinco@us.es (C.S.); 2 Department Ciencias Agroforestales, Universidad de Sevilla, Escuela Técnica Superior de Ingeniería Agronómica, Carrera de Utrera Km1, 41013 Sevilla, Spain; mcorell@us.es; 3 Unidad Asociada al CSIC de Uso Sostenible del Suelo y el Agua en la Agricultura (US-IRNAS), Crta. De Utrera Km 1, 41013 Sevilla, Spain; 4 Department Química Analítica, Facultad de Farmacia, Universidad de Sevilla, 41012 Sevilla, Spain; 5 Carrera de Ingeniería en Biotecnología de los Recursos Naturales, Universidad Politécnica Salesiana, Sede Quito, Campus El Girón, Av. 12 de Octubre, 170517 Quito, Ecuador; ecoyagoc@ups.edu.ec

**Keywords:** antioxidants, edible flowers, functional foods, petals, phytochemicals, retinol activity equivalents

## Abstract

Flowers have been used for centuries in decoration and traditional medicine, and as components of dishes. In this study, carotenoids and phenolics from 125 flowers were determined by liquid chromatography (RRLC and UHPLC). After comparing four different extractants, the carotenoids were extracted with acetone: methanol (2:1), which led to a recovery of 83%. The phenolic compounds were extracted with 0.1% acidified methanol. The petals of the edible flowers *Renealmia alpinia* and *Lantana camara* showed the highest values of theoretical vitamin A activity expressed as retinol activity equivalents (RAE), i.e., 19.1 and 4.1 RAE/g fresh weight, respectively. The sample with the highest total phenolic contents was *Punica granatum* orange (146.7 mg/g dry weight). It was concluded that in most cases, flowers with high carotenoid contents did not contain high phenolic content and vice versa. The results of this study can help to develop innovative concepts and products for the industry.

## 1. Introduction

Flowers have long held an important place in human societies. They have been used for ornamental purposes as well as in diverse dishes, mainly due to their appealing and diverse colors [1]. In addition, flowers have been used in traditional medicine [2]. More specifically, the use of flowers in the diet or as medicine dates back at least to 4000 BC, as documented in the Mesopotamic and Egyptian cultures [3]. Their traditional use in other cultures (Roman, Greek, Chinese, Indian, and European) is also well-known [4].

In recent years, there has been a growing interest in the study from different points of view of the health-promoting secondary metabolites present in flowers, including carotenoids and phenolics [5,6,7]. Indeed, the study of agronomic practices that can enhance the levels of these compounds in flowers or non-conventional technologies for their extraction are timely topics [8,9]. Carotenoids (carotenes and xanthophylls) are widespread and versatile compounds in nature, where they are important in processes including photosynthesis, the communication within and between species, the protection against oxidizing agents, and the modulation of membrane properties [10]. They are responsible for the red, yellow and orange colors of many flowers [11], which are important for pollination [12]. One of the main differences between carotenoids relative and other bioactive compounds is that some of them can be converted into vitamin A, which is an essential micronutrient. Apart from their key role in combating vitamin A deficiency and as natural food colors, carotenoids are important in health promotion. In fact, these compounds can help to enhance the immune system and reduce the risk of developing some diseases, including cancers (prostate, breast, cervical, ovarian, and colorectal), cardiovascular disease, bone, skin, and eye disorders. Although the possible health-promoting actions of carotenoids are commonly attributed to their antioxidant capacity, they can act through other mechanisms, such as the modulation of signaling pathways (with antioxidant, detoxifying and antiinflamatory effects), the enhancement of intercellular communication, or the protection against light [13]. Due to their versatility, carotenoids have applications not only in the food industry (colorants, ingredients, source of vitamin A), but also in cosmetics [14], feeds [15], pharmaceuticals [16], and even as textile dyes [17].

Phenolic compounds are, like carotenoids, widespread compounds in nature in general and in plants in particular. They can be categorized as extractable or non-extractable. Phenolic acids (benzoic and hydroxycynnamic acids), flavonoids (flavonols, flavones, flavanols, isoflavones, flavanones, and anthocyanidins), estilbenes, extractable proanthocyanidins, and hydrolyzed tannins belong to the first group. Non-extractable proanthocyanidins (or condensed tannins) and hydrolysable phenolics are groups of non-extractable phenolics [18]. These compounds also elicit great interest due to their health-promoting activities, which are usually attributed to antioxidant activity, although there is also evidence that they could exhibit antiviral, anticarcinogenic, antiinflamatory or antimicrobial activities, among others [19].

Depending on their fitness for human consumption, flowers are classified as edible or inedible, which depends on factors including the levels of inherent toxic compounds and/or those of fertilizers, herbicides or pesticides that can be dangerous for human health [20]. Edible flowers are normally used as flavor enhancers, relishes, vegetables, or dish decorations [21]. Common examples are roses (Rosa spp.) in Italy, dandelions (Taraxacum officinale) in Europe, and violets (Viola tricolor) in USA [22]. All in all, there is an increased market demand for edible flowers [1], increasing the need to further study the presence of compounds with nutritional interest in them. In this context, the objective of this study was to evaluate the carotenoids and phenolics of 125 flowers through liquid chromatography,

## 2. Materials and Methods

### 2.1. Reagents and Standards

The methanol, hexane, acetone, petroleum ether, dichloromethane, and hydrochloric acid were of analytical grade and were purchased from Labscan (Dublin, Ireland). The HPLC-grade methanol, HPLC-grade acetonitrile, HPLC–grade ethyl acetate, formic acid, sodium chloride, and potassium hydroxide were obtained from Panreac (Barcelona, Spain). The β-Carotene, all-*trans*-β-apo-8′-carotenal, α-carotene, phytoene, violaxanthin, lutein, β-cryptoxanthin, and lycopene were purchased from Sigma-Aldrich (Taufkirchen, Germany) and the antheraxanthin from DHI (Hørsholm, Denmark). The lutein epoxide, luteoxanthin, zeinoxanthin, 9-*cis*-antheraxanthin, 9-*cis*-violaxanthin, 13-*cis*-violaxanthin, and 9-*cis*-lutein were obtained as described elsewhere [23,24,25,26,27]. The gallic acid, *p*-hydroxybenzoic acid, syringic acid, caffeic acid, *m*-coumaric, *p*-coumaric, chlorogenic acid, ferulic acid, naringin, naringenin, ethyl galate, quercetin, kaempferol, crisin, vanillic acid, and myricetin were purchased from Sigma-Aldrich (Madrid, Spain). The quercitrin was obtained from Extrasynthese (Genay, France). All the aqueous solutions were prepared with purified water in a NANOpure Dlamond^TM^ system (Barnsted Inc., Dubuque, IO, USA).

### 2.2. Plant Materials

The petals of one hundred twenty-five fresh flowers from 52 different families and 102 species were collected from a botanical garden (Real Jardín Botánico de Córdoba, Córdoba, Spain) and local greenhouses in Madrid and Seville (Spain). These places ensure the traceability in the growth of the floral species by providing identification. After measuring the color of the petals, the samples were freeze-dried (Cryodos-80, Telstar, Terrasa, Spain) and the humidity was calculated.

### 2.3. Color Analysis

The colors were measured using a CM-700d colorimeter (Minollta, Japan). Illuminant D65 and 10° observer were considered as references. The color parameters corresponding to the uniform color space CIELAB were obtained. The categorization of the samples by color (white, yellow, orange, red, pink, lilac and blue) was performed considering clusters of points in the a*b* plane. Thus, the samples were separated into three groups. Group A included white, yellow, and orange flowers, group B contained red and pink flowers, and group C included lilac and blue flowers. The color of some flowers could not be assessed instrumentally because of their small sizes.

### 2.4. Analysis of Carotenoids

#### 2.4.1. Extraction and Saponification

The micro-extractions were performed under dim light and in triplicate. The best extraction mixture was selected after evaluating different extraction mixtures (hexane: acetone (*v/v*) (1:1), methanol: acetone: dichloromethane (*v/v/v*) (1:1:2), acetone: methanol (*v/v*) (2:1), and ethyl acetate: methanol: petroleum ether (*v/v/v*) (1:1:1)). For this purpose, the petals of Calendula × hybrid were used. Approximately 20 mg of homogenized freeze-dried powder was mixed with 1 mL of the appropriate solvent mixture and then vortexed, sonicated for 2 min and centrifuged at 14,000× *g* for 3 min. After recovering the colored fraction, the extraction was repeated with aliquots of 500 μL of the solvent mixture until color exhaustion. The organic colored fractions were combined and evaporated to dryness in a vacuum concentrator at a temperature below 30 °C. Calendula × hybrid is known to possess high amounts of esterified carotenoids, so the extracts were de-esterified by saponification [28]. For this purpose, the dry extracts were re-dissolved in 500 μL of methanolic potassium hydroxide (30%, *w/v*) and the mixtures were stirred for one hour in a nitrogen atmosphere at 25 °C. Next, 500 μL of dichloromethane and 800 μL of 5% aqueous NaCl (*w/v*) were added. The samples were vortexed and centrifuged at 14,000× *g* for 3 min and then the aqueous phase was removed. The carotenoid-containing phase was washed with water until neutrality of the wastewater. The colored phase was evaporated to dryness in a vacuum concentrator at a temperature below 30 °C and stored in a nitrogen atmosphere at −20 °C until the analysis.

The extraction mixture leading to the highest recovery of carotenoids was selected for the extraction of carotenoids from all the samples. All-*trans*-β-apo-8´-carotenal was used as an internal standard.

#### 2.4.2. Spectrophotometric Analysis

The total carotenoid contents (TCC) of petroleum ether extracts of each flower were quantified by spectrophotometry by considering the absorbance reading at 450 nm and the molar absorptivity value of β-carotene in the solvent (εmol = 2592). The results were reported as μg/g dry weight (DW) [29].

#### 2.4.3. Rapid Resolution Liquid Chromatography (RRLC) Analysis

The dry extracts were re-dissolved in 20 μL of ethyl acetate prior to their analysis by RRLC. The analysis was carried out using the method reported by [30] on an Agilent 1260 system equipped with a diode-array detector and a C18 Poroshell 120 column (2.7 μm, 5 cm × 4.6 mm) (Agilent, Palo Alto, CA, USA). The injection volume was 5 μL, the flow rate was 1 mL/min, and the temperature of the column was set at 30 °C. A mobile phase consisting of acetonitrile, methanol, and ethyl acetate was used with a linear gradient elution [30]. The chromatograms were monitored at 285, 350, and 450 nm for the quantification of phytoene, phytofluene, and the rest of the carotenoids (lutein epoxide, luteoxanthin, antheraxanthin, violaxanthin, lutein, *cis*-antheraxanthin, lycopene, zeinoxanthin, β-cryptoxanthin, β-carotene, and α-carotene), respectively. UV–Vis spectra were recorded from 250 to 750 nm. The individual carotenoids were identified with their corresponding standards and quantified using external calibration curves made with them whenever possible. The limits of detection (LOD) and quantification (LOQ) were calculated as three and ten times, respectively; the relative standard deviation of the analytical blank values were calculated from the calibration curve, using Microcal Origin ver. 3.5 software (OriginLab Corporation, Northampton, MA, USA). The LODs and LOQs ranged from 0.002 µg in phytoene to 0.070 µg in lycopene and from 0.007 µg in phytoene to 0.232 µg in lycopene, respectively. The LOD and LOQ were established on the basis of signal to noise (S/N) ratio of 3 and 10, respectively. The samples were analyzed in duplicate with double sample injection. The concentrations were expressed in μg/g DW and the TCC contents were calculated by adding up all the individual carotenoids.

### 2.5. Analysis of Phenolic Compounds

#### 2.5.1. Extraction

The protocol described by [31] was adapted for the extraction of smaller amounts of samples. Briefly, 1.5 mL of 0.1% acidified methanol was added to approximately 50 mg of freeze-dried petals, and the mixture was vortexed, sonicated for 2 min, and centrifuged at 4190× *g* for 7 min and at 4 °C; the supernatant was collected and the residue was submitted to the same extraction process twice with only 0.5 mL of the acidified methanol. The combined supernatant was stored at −20 °C until the analysis.

#### 2.5.2. Spectrophotometric Analysis

The extract obtained was used for the determination of the total phenolic content (TPC) using the Folin–Ciocalteu assay, as described by [31], with slight modifications. Briefly, 50 μL of extract, 0.25 mL of Folin-Ciocalteu reagent, 0.75 mL of a solution of sodium carbonate (20%), and 3.95 mL of distilled water were mixed and left to stand for 2 h for the reaction to take place. Gallic acid was employed as a calibration standard and the absorbance was read at 765 nm with a Hewlett-Packard UV-vis HP8453 spectrophotometer (Palo Alto, CA, USA). The results were expressed as mg of equivalents of gallic acid per g of dry weight (mg GAE/ g DW) and allowed to define the injection volumes for the quantification by Ultra-High Performance Liquid Chromatography (UHPLC).

#### 2.5.3. Ultra-High Performance Liquid Chromatography (UHPLC) Analysis

Prior to the injection, the extracts were concentrated to dryness, re-dissolved in 20 μL of 0.01% formic acid, and centrifuged at 4190× *g* for 7 min and at 4 °C. The UHPLC method was previously reported by [31]. An Agilent 1290 chromatograph equipped with a diode-array detector (Agilent Technologies, Palo Alto, CA, USA) set between 220 and 500 nm and an Eclipse Plus C18 column (1.8 um, 2.1 × 5 mm) were used. The column was kept at 30 °C, the injection volumes were in a range between 0.3 and 1.5 μL, the flow rate was 1 mL/min, and a linear gradient was used. Open lab ChemStation software was used for data acquisition and processing. The identification of the phenolic compounds was performed through a comparison of their retention times and UV-vis spectra, within the range 250–750 nm, with those of the available standards [31]. The chromatograms were monitored at 280 for the benzoic acids, hydroxycinnamic acids, flavones, and flavanones, and at 320 nm for the flavonols. Their quantification was carried out using external calibration curves of each of the compounds analyzed. The LODs and LOQs ranged from 0.006 µg in chlorogenic acid to 0.012 µg in *p*-hydroxybenzoic acid and 0.014 µg in chlorogenic acid to 0.041 µg in *p*-hydroxybenzoic acid, respectively. The LOD and LOQ were established on the basis of signal-to-noise (S/N) ratios of 3 and 10, respectively. The samples were analyzed in duplicate with double sample injection. The TPC was calculated by adding up all the individual phenolics.

### 2.6. Statistical Analysis

All the experiments were performed in triplicate with double injection, and the results were expressed as mean ± standard deviation (SD). The mean separation was made via Tukey’s test. Differences were considered statistically significant for *p* values ≤0.01. The statistical analysis was performed using the STATGRAPHICS Centurion XVII software.

## 3. Results

### 3.1. Color Parameters and Other Characteristics

The color parameters, humidity values, and culinary uses of the flowers are presented in Table 1, Table 2 and Table 3. 

### 3.2. Carotenoids

#### Selection of the Extraction Solvents

**Figure 1:**
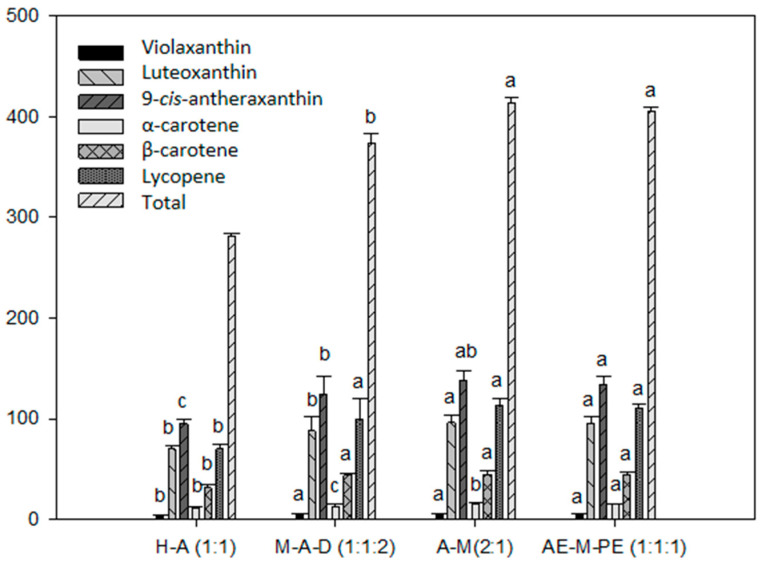
Carotenoid content recoveries (mg/100 g DW) after extraction of Calendula × hybrid using four different extraction solvents. H-A (1:1), hexane: acetone (*v/v*) (1:1); M-A-D (1:1:2), methanol: acetone: dichloromethane (*v/v/v*) (1:1:2); A-M (2:1), acetone: methanol (*v/v*) (2:1); AE-M-PE (1:1:1), ethyl acetate: methanol: petroleum ether (*v/v/v*) (1:1:1). Different letters among bars of the same carotenoid indicate significant differences by ANOVA test (*p* < 0.01).

Four different extraction solvents were tested for the extraction of carotenoids in Calendula × hybrid (Figure 1). Acetone: methanol (*v/v*) (2:1) and ethyl acetate: methanol: petroleum ether (*v/v/v*) (1:1:1) showed the highest carotenoid extraction yields and there was no statistically significant difference between the two mixtures. The recovery of carotenoids obtained with this mixture, using all-*trans*-β-apo-8′-carotenal as internal standard, was 83%.

**Figure 2:**
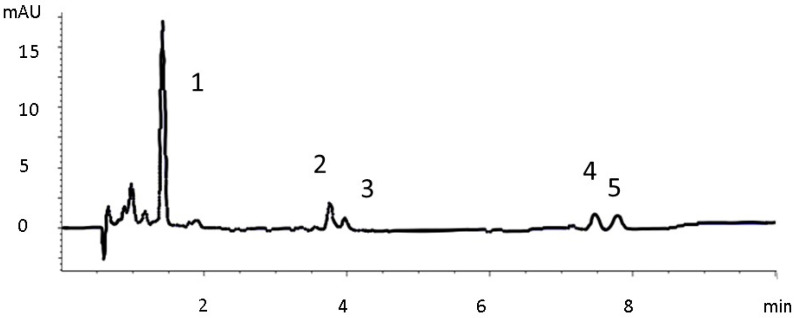
Chromatogram of *Lavandula angustifolia* at 450 nm (C18 column). 1. Lutein; 2. Zeinoxanthin; 3. β-Cryptoxanthin; 4. α-Carotene; 5. β-Carotene.

**Figure 3:**
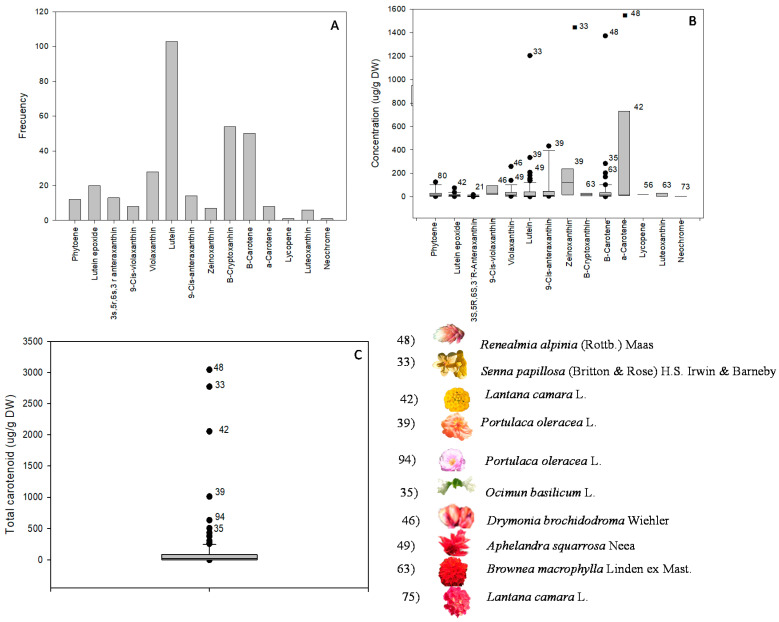
Frequency, mean contents, and standard deviations of carotenoids and major sources. Frequency (**A**), mean contents of individual carotenoids (**B**), total carotenoid content (**C**), and list of species with high concentrations of carotenoids. Number within the figures represent the sample under study.

In addition, quantitative data on individuals and TCC, assessed by liquid chromatography, are presented in Table 4, Table 5 and Table 6. An example of the resulting chromatogram is presented in Figure 2, and the frequency, mean contents, and standard deviations of carotenoids and major sources are presented in Figure 3, sections A, B, and C.

### 3.3. Phenolic Compounds

**Figure 4:**
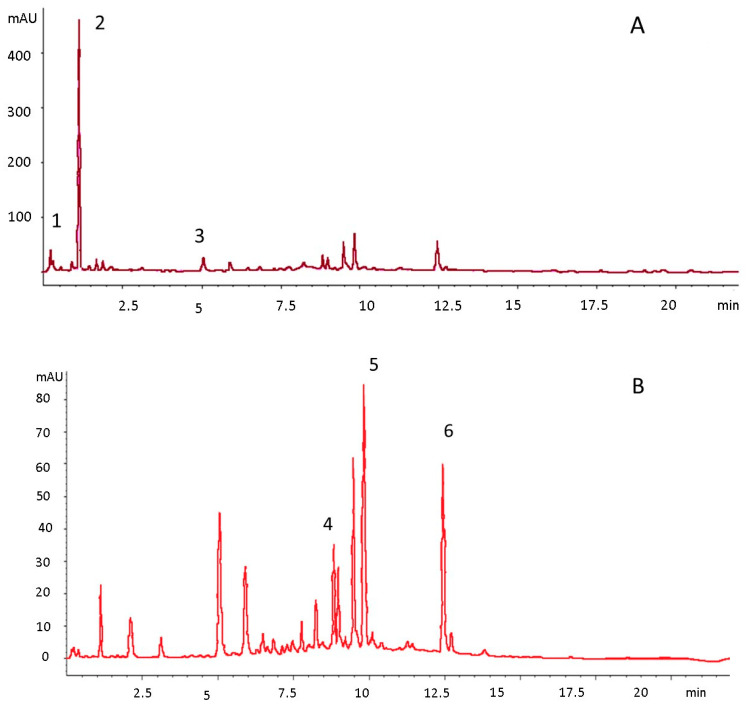
Chromatogram of lilac *Catharanthus roseus* phenolics at 280 nm (**A**) and 320 nm (**B**). 1. *p*-Hydroxybenzoic acid, 2. *m*-Coumaric acid, 3. Chlorogenic acid, 4. Quercitrin, 5. Quercetin, 6. Kaempferol.

**Figure 5:**
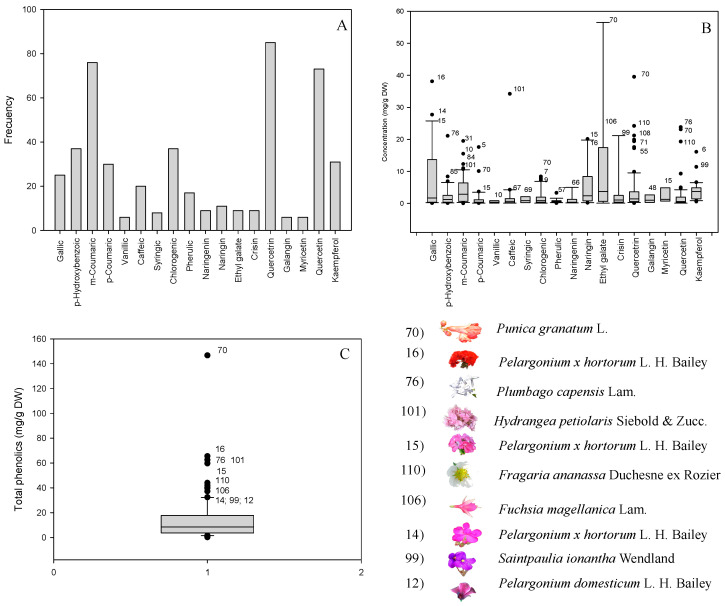
Frequency, mean contents and standard deviations of phenolics and major sources. Frequency (**A**), mean contents of individual phenolics (**B**), total phenolics content (**C**), and list of species with high concentrations of phenolics. Number within the figures represent the sample under study.

The quantitative data on individuals and TPC assessed by chromatographic analysis are presented in Table 7, Table 8 and Table 9. In addition, an example of the resulting chromatogram is presented in Figure 4 and Figure 5 sections A, B, and C show the frequency, mean contents, and standard deviations of the phenolics and major sources.

## 4. Discussion

### 4.1. Color Parameters and Other Characteristics

The great majority of the flowers were edible (*n* = 111, i.e., 89%); 70% of the families studied (52 families) included edible flowers. For example, the families Asteraceae and Lamiaceae contained six and seven edible species, respectively [20]. Concerning their uses, the most frequent were in salads (31.3% of the total use of the flowers) and infusions (28.9%), followed by teas (15.7%), desserts (13.3%) and others, including as garnishes and colorants (Table 1, Table 2 and Table 3). The different culinary uses of flowers depend to some extent on their size, shape, and color, as suggested by other authors [4]. These characteristics varied considerably among the samples surveyed in the present study. Different shapes were found, such as tubular (e.g., *Russelia equisetiformis* Schltdl. Et Cham.), bilabial (e.g., *Rosmarinus officinalis* L.), flared (e.g., *Punica granatum* L.), and flowers that form part of a cluster (e.g., *Plantago major* L., *Salvia splendens* Sellow ex Schylt., *Vitex agnus-castus* L., *Allium schoenoprasum* L., and *Lantana camara* L.). On the other hand, the flowers showed a great variety of colors (Table 1, Table 2 and Table 3), such as white (e.g., *Portulaca oleracea* L.), yellow (e.g., *Anthemis tinctoria* L.), orange (e.g., *Punica granatum* L.), pink (e.g., *Diantuhus caryophyllus* L.), red (e.g., *Pelargonium × hortorum*), lilac (e.g., *Petunia hybrid*), and blue (e.g., *Lavandula angustifolia* Mill.). The color parameters ranged between 14.2 and 87.1, −9.2 and 57.2, −25.7 and 88.6, 2.9 and 89.9, and 3.4 and 359.6 for L* (lightness), a* (ranging from green to red), b* (ranging from blue to yellow), C*_ab_ (chroma, the quantitative expression of color), and *h_ab_* (hue angle, the qualitative expression of color), respectively. The variety of colors found in the petals of flowers under study can be explained by the different contents of carotenoids and phenolics, which are usually the main contributors to the color of these structures [34,35]. 

The humidity of the petals ranged between 54.5 and 99.7%, a wider interval compared to that recently reported by other authors (70 and 95%) [4].

### 4.2. Carotenoids

#### 4.2.1. Selection of the Extraction Solvents

Regarding the quantification of carotenoids in flowers, there are several studies that use different extraction solvents; however, the mixtures acetone: methanol (2:1) and ethyl acetate: methanol: petroleum ether (1:1:1) in this study presented the highest extraction percentage. Acetone: methanol (2:1) was selected as the extraction solvent for the studied flowers due to its slightly higher yield and its simplicity of preparation.

#### 4.2.2. Carotenoid Levels

At this point it is important to notice that saponification, which simplifies the identification of carotenoids, has the disadvantage that it leads to carotenoid losses [30], so the information provided must be interpreted with this in mind. This fact has been observed in the TCC levels of red and lilac flowers of *Catharanthus roseus* (3.7 µg/g DW and not detectable, respectively) and *Pelargonium × hortorum* (3.5 µg/g DW and not detectable, respectively). Although the TCC levels measured in non-saponified extracts by spectrophotometry showed values of 185, 132, and 100 µg/g DW, respectively, no individual carotenoids were detected by RRLC after the saponification of the extracts (data not shown).

On the other hand, flowers of the same family but different species presented different profiles in most cases. At this point it is important to notice that the profiles of the secondary metabolites of plants in general and carotenoids and phenolics in particular are dependent on different factors, including genotype as one of the most important, along with ambient/seasonal (light quality and quantity, temperature), and agronomic factors (irrigation, fertilization, etc.), among others [36,37,38].

Lutein (31.7%), β-cryptoxanthin (16.6%), and β-carotene (15.4%) were the most frequent carotenoids (Figure 3, section A). These three carotenoids are, along with zeaxanthin, α-carotene, lycopene, phytoene, and phytofluene, the major carotenoids in human tissues and fluids, all of which are thought to promote health [13]. All of them, except phytofluene, were identified in the set of samples, as well as others not reported in humans, such as lutein epoxide, antheraxanthin, violaxanthin, zeinoxanthin, luteoxanthin, and neochrome (Figure 3, section A).

Figure 3, section B, presents the mean contents and standard deviations of the individual carotenoids. The levels of the colorless carotenoid phytoene ranged between 2.8 (*Trifolium cernuum)* and 126.4 µg/g DW (*Guzmania hybrid*). The concentrations of lutein ranged from 0.7 to 1204.0 µg/g DW. The best source by far was *Senna papillosa* yellow (1204.0 µg/g DW), followed by *Portulaca oleracea* yellow (334.9 µg/g DW) and *Aphelandra squarrosa* red (209.0 µg/g DW), in descending order. The levels of lutein epoxide ranged from 2.9 to 75.8 µg/g DW. The highest amounts were found in *Lantana camara* yellow (75.8 µg/g DW), *Mentha suaveolens* white (38.5 µg/g DW), *Solanum lycopersicum* yellow (31.9 µg/g DW), and *Mentha × piperita* lilac (23.4 µg/g DW). The concentrations of luteoxanthin fell in an interval of 1.1–98.7 µg/g DW, and the main sources were *Brownea macrophylla* red (98.7 µg/g DW), *Mentha suaveolens* white (6.9 µg/g DW), and *Mentha × piperita* lilac (5.9 µg/g DW). On the other hand, the concentrations of antheraxanthin ranged from 1.8 to 18.3 µg/g DW. *Capsicum annuum* white (18.3 µg/g DW), *Campanula shetleri* white (11.4 µg/g DW), and *Rosa hybrid* pink (9.6 µg/g DW) were the flowers with the highest contents. The 9-*Cis*-antheraxanthin concentration values fluctuated between 5.4 and 433.4 µg/g DW. The highest levels were detected in *Portulaca oleracea* yellow (433.4 µg/g DW) and pink (355.2 µg/g DW) petals. The concentrations of violaxanthin ranged from 4.0 to 258.8 µg/g DW. *Drymonia brochidodroma* orange (258.8 µg/g DW), *Aphelandra squarrosa* red (140 µg/g DW), and *Senna papillosa* yellow (86.0 µg/g DW) were the best sources. The concentrations of the carotenoid identified as zeinoxanthin varied between 4.5 and 1311.9 µg/g DW. The best sources were *Senna papillosa* yellow (1311.9 µg/g DW) and, to a much lesser extent, *Portulaca oleracea* yellow (239.8 µg/g DW). The levels of the provitamin A carotenoid β-cryptoxanthin ranged from 4.2 to 33.4 µg/g DW; the highest amounts were found in *Brownea macrophylla* red (33.4 µg/g DW) and *Lavandula angustifolia* blue (19.3 µg/g DW). The amounts of the provitamin A carotenoid α-carotene were in the interval of 12.3–1451.9 µg/g DW. *Renealmia alpinia* orange (1451.9 µg/g DW) and, to a lesser extent, *Lantana camara* yellow (731.5 µg/g DW) and *Spathiphyllum montanum* white (82.0 µg/g DW) stood out as the main sources.

Britton and Khachik proposed a criterion through which to classify food sources according to their carotenoid content expressed in mg/100 g fresh weight. According to this criterion, the contents of a specific carotenoid can be classified as low (0–0.1 mg/100 g), moderate (0.1–0.5 mg/100 g), high (0.5–2 mg/100 g), or very high (>2 mg/100 g).

Using this criterion to categorize carotenoid sources, the petals with high (0.5–2 mg/100 g) or very high (>2 mg/100 g) carotenoid levels are *Renealmia alpinia* (15.0 mg/100 g FW), *Senna papillosa* (4.5 mg/100 g FW), *Sophora japonica*, *Brownea macrophylla* (2.6 mg/100 g FW) (β-carotene), *Tecoma capensis* (0.5 mg/100 g FW) (β-cryptoxanthin), *Senna papillosa* (31.8 mg/100 g FW), *Aphelandra squarrosa* (5.6 mg/100 g FW), *Portulaca oleracea* (4.7 mg/100 g FW) (lutein), *Lantana camara* (1.7 mg/100 g FW)(zeaxanthin), and *Lantana camara* (0.6 mg/100 g FW) (phytoene).

On the other hand, the maximum daily intakes of carotenoids reported in recent reviews were 4.1 (lutein + zeaxanthin), 1.4 (β-cryptoxanthin), 2.4 (α-carotene), 8.8 (β-carotene), 9.4 (lycopene), 2.0 (phytoene), and 0.7 mg (phytofluene) [13]. These intakes could be obtained with 87.2 g FW of *Portulaca oleracea* (lutein + zeaxanthin), 280 g FW of *Tecoma capensis* (β-cryptoxanthin), 15.2 g FW of *Renealmia alpinia* (α-carotene), 58.7 g FW of *Renealmia alpinia* (β-carotene), and 153.8 g FW of *Guzmania hibrid* (phytoene). These data indicate that the consumption of just a few grams of petals of some flowers (for instance, *Portulaca oleracea* or *Renealmia alpinia*) can be useful to increase considerably the intakes of health-promoting carotenoids.

The petals of the edible flowers *Renealmia alpinia* (15.0 mg/100 g FW of β-carotene and 15.8 mg/100 g FW of α-carotene) and *Lantana camara* (0.6 mg/100 g FW of β-carotene and 8.6 mg/100 g FW of α-carotene) showed the highest values of provitamin A carotenoids. The values of vitamin A activity of the samples can be expressed in terms of retinol activity equivalents (RAE), considering the equivalences 1 RAE = 12 μg of all-*trans*-β-carotene = 24 μg of other provitamin A carotenoids [39]. Thus, the RAE per gram of fresh weight of *Renealmia alpinia* and *Lantana camara* are 19.1 and 4.1, respectively. Given that 1 RAE equals two retinol equivalent (RE), it can be estimated that 10 g of fresh flowers from *Renealmia alpinia* would provide 381.2 retinol equivalents (RE), which is approximately half the daily recommendation of vitamin A for adults (750 RE/day) by FAO and OMS [40].

The TCC values obtained as the sum of the levels of individual carotenoids ranged from 1.7 (*Aloysia citriodora,* pink) to 3044.7 µg/g DW (*Renealmia alpinia*, orange). The flowers with the highest TCC levels were *Renealmia alpinia* orange (3044.7 µg/g DW), *Senna papillosa* yellow (2772.2 µg/g DW), *Lantana camara* yellow (2056.0 µg/g DW), and *Portulaca oleracea* yellow (1012.4 µg/g DW). The TCC content of *R. alpinia* was outstanding, as it was 23 times higher than the mean TCC of all the flowers. Foods with high or very high carotenoid levels are: green vegetables, apricot, carrot, mango, palm oil, buriti, and sweet potato (β-carotene); persimmon, pitanga, papaya, pumpkin, and tangerines (β-cryptoxanthin); green vegetables, pumpkin, sastra, and egg yolk (lutein); Chinese wolfberry, sastra, corozo, sapote, quince, orange, and red peppers (zeaxanthin); tomato, watermelon, red grapefruit, and papaya (lycopene); tomato, apricot, red pepper, carrot, and red grapefruit (phytoene and phytofluene) [5,15,17,28].

### 4.3. Phenolic Compounds

The most frequent phenolic compounds in the set of flowers evaluated were *m*-coumaric acid (a phenolic acid), quercitrin, and quercetin (flavonoids) (Figure 5, section A), which agrees well with the information reported by other authors indicating that phenolic acids and flavonoids are the predominant phenolic compounds in flowers [41]. On the other hand, values between 4.83 and 222.00 mg GAE/g DW of total phenolics have been described in 23 edible flowers elsewhere [4].

Flowers of the same species with different colors showed different profiles of phenolics, as opposed to what was observed in the case of carotenoids. Flowers of different species also exhibited different phenolic patterns. This may have been due to the fact that, as already mentioned, the contents of phenolic compounds and other secondary metabolites in plants are dependent on genetic factors, as well as climatic and agronomic conditions, among others [37,38].

In addition, the influence of different methods on the extraction efficiency of different compounds (in this case not only phenolics but also carotenoids), and therefore on their, quantification must be taken into account.

#### 4.3.1. Benzoic Acids

Gallic acid displayed ranges between 0.1 and 38.1 mg/g DW. Pelargonium × hortorum red (38.1 mg/g DW), pink (24.5 mg/g DW), lilac (27.7 mg/g DW), and *Pelargonium domesticum* lilac (18.6 mg/g DW) were the samples with the highest contents. The content of *p*-Hydroxybenzoic acid ranged from 0.1 to 21.1 mg/g DW. The highest values were found in *Plumbago auriculata* white (21.1 mg/g DW), *Chlorophytum comosum* white (8.4 mg/g DW), *Dahlia coccinea* yellow (6.9 mg/g DW), and *Vitex agnus castus* lilac (6.5 mg/g DW). The *m*-coumaric acid values showed ranges between 0.04 and 19.5 mg/g DW. *Verbena × hybrid* pink (19.5 mg/g DW), *Dianthus caryophyllus* red (15.5 mg/g DW), *Vitex agnus-castus* lilac (15.5 mg/g DW), and *Hydrangea petiolaris* pink (12.3 mg/g DW) exhibited the most significant *m*-coumaric acid concentrations. The *p*-coumaric acid values oscillated between 0.1 and 17.6 mg/g DW. *Catharanthus roseus* red (17.6 mg/g DW) and *Punica granatum* orange (10.1 mg/g DW) were the samples surveyed with the highest values of *p*-coumaric acid. The levels of vanillic acid fell in an interval of 0.1–1.6 mg/g DW. This compound was detected only in a few species, such as *Dianthus caryophyllus* red (1.6 mg/g DW), *Vitex agnus castus* lilac (0.6 mg/g DW), *Nerium oleander* pink (0.5 mg/g DW), *Celosia argentea* red (0.1 mg/g DW), and *Aglaonema commutatum* yellow (0.1 mg/g DW). The Syringic acid totals ranged from 0.1 to 3.0 mg/g DW. *Lagerstroemia indica* pink (3.0 mg/g DW) and *Pelargonium domesticum* lilac (2.4 mg/g DW) were the flowers with the highest concentrations.

#### 4.3.2. Hydroxycinnamic Acids

The concentrations of caffeic acid ranged from 0.1 to 34.2 mg/g DW. *Hydrangea petiolaris* pink (34.2 mg/g DW), *Salvia splendens* red (4.3 mg/g DW), and *Convolvus althaeoides* blue (4.2 mg/g DW) were the richest sources of this compound. The chlorogenic acid content ranged between 0.1 and 8.4 mg/g DW. Its main sources were *Punica granatum* orange (8.4 mg/g DW), *Nerium oleander* pink (7.9 mg/g DW) and red (7.3 mg/g DW), *Anthemis tinctoria* yellow (6.8 mg/g DW), and *Hydrangea petiolaris* pink (6.6 mg/g DW). The ferulic acid levels oscillated between 0.1 and 3.3 mg/g DW. *Convolvulus althaeoides* blue (3.3 mg/g DW), *Nerium oleander* pink (1.2 mg/g DW), *Petunia hybrida* lilac (0.9 mg/g DW), and *Dahlia pinnata* yellow (0.9 mg/g DW) were its main sources.

#### 4.3.3. Flavonols

The quercitrin concentrations ranged from 0.1 to 39.5 mg/g DW. The main sources of this flavanol were *Punica granatum* orange (39.5 mg/g DW), *Fragaria × ananassa* white (24.2 mg/g DW), *Plantago major* yellow (21.2 mg/g DW), *Hibiscus syriacus* lilac (19.9 mg/g DW), and *Gypsophila paniculata* white (19.4 mg/g DW). The quercetin levels oscillated between 0.1 and 23.8 mg/g DW. Its major sources were *Plumbago auriculata* white (23.8 mg/g DW), *Punica granatum* orange (23.1 mg/g DW), and *Fragaria × ananassa* white (19.3 mg/g DW). Myricetin was present in the set of samples at concentrations in the range 0.4 - 8.2 mg/g DW and was only detected in *Pelargonium × hortorum* pink (8.2 mg/g DW) and red (3.8 mg/g DW), *Dianthus caryophyllus* pink (1.5 mg/g DW), *Pelargonium domesticum* lilac (1.0 mg/g DW)*,* and *Fallopia aubertii* lilac (0.8 mg/g DW) and yellow (0.4 mg/g DW). The levels of kaempferol in the set of flowers were in an interval of 0.6–16.1 mg/g DW. *Catharanthus roseus* lilac (16.1 mg/g DW), *Saintpaulia ionantha* blue (11.4 mg/g DW), and *Rosa hybrid* red (6.6 mg/g DW) were the best sources.

#### 4.3.4. Flavones

Crisin was detected only in a few samples, at concentrations between 0.1 and 21.2 mg/g DW. *Saintpauli ionantha* blue (21.2 mg/g DW), *Cuphea hyssopifolia* pink (11.3 mg/g DW), and *Lantana camara* (Verbenaceae family) white (3.4 mg/g DW) displayed the highest values.

#### 4.3.5. Flavanones

The naringin values were between 0.2 and 20.1 mg/g DW and *Pelargonium × hortorum* pink (20.1 mg/g DW) and red (18.6 mg/g DW) and *Pelargonium peltatum* red (8.4 mg/g DW) exhibited the highest values.

#### 4.3.6. Total Phenolic Compounds

TPC demonstrated ranges between 0.2 (*Ocimum basilicum* white) and 146.7 mg/g DW (*Punica granatum* orange) (Figure 3, section C). The TPC in *Punica granatum* was noteworthy as it was 15 times higher than the mean of TPC in the entire set of flowers. *Punica granatum* orange (147.0 mg/g DW), *Pelargonium × hortorum* red (65.5 mg/g DW) and pink (59.7 mg/g DW), *Hydrangea petiolaris* pink (62.6 mg/g DW), and *Plumbago auriculata* white (62.5 mg/g DW) showed the highest values of TPC in the flowers under study. Other authors found similar values of TPC in *Pelargonium × hortorum* (i.e., 50.4 mg GAE/g DW using the same humidity as this study) [42]. *Anthemis tinctoria* with chlorogenic acid (6.8 mg/g DW) as the major compound and 11.5 mg/g DW of TPC, *Mirabilis jalapa* with *p*-hydroxybenzoic acid (4.5 mg/g) and 9.5 mg/g of TPC, *Limonium sinuatum* with kaempferol (1.0 mg/g DW) and 1.8 mg/g DW of TPC, *Euonymus japonicus* with quercitrin (0.8 mg/g DW) and 1.9 mg/g DW of TPC, and *Gardenia jasminoides* with ferulic acid and naringin (0.6 mg/g DW both) and 1.9 mg/g DW of TPC are used as food additives and natural colorants [43,44,45]. Other authors have reported values of 9.06 mg/100 g DW, 19.06 mg/100g DW, and 190.8 mg GAE/100 g DW for gallic acid, quercetin, and total phenolic, respectively, in *Gardenia jasminoides* [46]. Furthermore, other authors reported that *Mirabilis jalapa* is a good source of flavonoids and phenolic acids (ferulic acid and caffeic acid as major compounds) [47]. The aforementioned flowers, despite being used as coloring agents, did not stand out for their TPC in the present study.

## 5. Conclusions

In this study, the carotenoids and phenolic compounds of 125 flowers were evaluated. Flowers with high TCC levels (assessed by liquid chromatography) were pinpointed, such as *Renealmia alpinia* orange (whose TCC levels was 23 times higher than the media), *Senna papillosa* yellow, *Lantana camara* yellow, and *Portulaca oleracea* yellow. The petals of the edible flowers *Renealmia alpinia* and *Lantana camara* stood out for their high content of provitamin A carotenoid. The main sources of the different carotenoids detected were Guzmania hybrid (phytoene), *Senna papillosa* (lutein), *Renealmia alpinia* (β-carotene), *Lantana camara* yellow (lutein epoxide), *Brownea macrophylla* red (luteoxanthin), *Capsicum annuum* white (antheraxanthin), *Portulaca oleracea* yellow and pink petals (9-*cis*-antheraxanthin), *Drymonia brochidodroma* orange (violaxanthin), *Senna papillosa* yellow (zeinoxanthin), *Brownea macrophylla* red (β-cryptoxanthin), and *Renealmia alpinia* orange (α-carotene).

Some petals are indeed highly concentrated sources of carotenoids, including provitamin A carotenoids. As an example, it has been estimated that 10 g fresh weight of the petals of *Renealmia alpinia* can provide 381.2 ER, which is approximately half the daily recommendation of vitamin A for adults (750 ER/day).

The samples with the highest TPC (assessed by liquid chromatography) were *Punica granatum* orange, Pelargonium *×* hortorum red and pink, and *Hydrangea petiolaris* pink and *Plumbago auriculata* white). The TPC in *Punica granatum* was approximately 15 times higher than the mean.

The main sources of the different phenolics detected were Pelargonium *×* hortorum red, pink, and lilac (gallic acid), *Plumbago auriculata* white (*p*-hydroxybenzoic acid), Verbena *×* hybrid pink (m-coumaric acid), *Catharanthus roseus* red (*p*-coumaric acid), *Dianthus caryophyllus* red and lilac (vanillic acid), *Lagerstroemia indica* pink (syringic acid), *Hydrangea petiolaris* pink (caffeic acid), *Punica granatum* orange (chlorogenic acid), *Convolvulus althaeoides* blue (ferulic acid), *Punica granatum* orange (quercitrin), *Plumbago auriculata* white (quercetin), Pelargonium *×* hortorum pink and red (myricetin), *Catharanthus roseus* lilac (kaempferol), *Saintpauli ionantha* blue (crisin), and Pelargonium *×* hortorum pink and red (naringin).

In summary, several petal matrices with interesting carotenoid or phenolic profiles (either by their total content or their levels of specific carotenoids or phenolics) were pinpointed. The information provided can help to design breeding programs aimed at producing flowers with increased carotenoid and/or phenolic levels and can be useful for the provision of natural colors for the agro-food or textile industries, as well as for the provision of beneficial compounds for the functional foods, nutricosmetics, and pharmaceutical industries.

## Figures and Tables

**Table 1:** Mean color parameter values, humidity, and culinary uses (according to Coyago, et al., (2017) [32] and The-Plant-List, (2019) [33]) of white, yellow, and orange flowers.

Samples	Family	Species	Common Name	Culinary Uses	Humidity (%)	L*	a*	b*	C*_ab_	h_ab_
White flowers										
1	*Araceae*	*Spathiphyllum montanum* Grayum	Peace flower	Non edible	89.767 ± 0.225	57.200 ± 6.263	−9.210 ± 0.676	24.800 ± 1.495	26.456 ± 0.709	110.333 ± 0.697
2	*Agavaceae*	*Chlorophytum comosum* (Thunb.) Jacques	Bad mother	Infusion	99.130 ± 0.701	na	na	na	na	na
3	*Amaryllidaceae*	*Agapanthus africanus* (L.) Hoffmanns	African lily	Infusion	76.112 ± 0.306	73.517 ± 0.804	−1.213 ± 0.211	4.557 ± 0.872	4.727 ± 0.798	105.413 ± 3.107
4	*Apiaceae*	*Coriandrum sativum* L.	Coriander	Salad, garrison	95.853 ± 0.027	na	na	na	na	na
5	*Apocynaceae*	*Nerium oleander* L.	Flower laurel	Non edible	79.493 ± 0.666	77.800 ± 3.581	−2.627 ± 0.316	7.637 ± 0.301	8.092 ± 0.268	109.291 ± 3.447
6	*Apocynaceae*	*Trachelospermum jasminoides* (Lind.) Len.	Starry jasmine	Infusion	95.800 ± 0.325	na	na	na	na	na
7	*Boraginaceae*	*Heliotropium arborescens* L.	Vanilla	Infusion	86.012 ± 0.152	na	na	na	na	na
8	*Brassicaceae*	*Matthiola incana* (L.) R. Br.	White violet	Infusion	99.718 ± 0.525	87.123 ± 0.023	−1.740 ± 0.017	21.623 ± 0.323	21.693 ± 0.124	94.558 ± 0.023
9	*Campanulaceae*	*Campanula shetleri* Heckard	Green bell	na	93.548 ± 0.011	67.010 ± 0.001	0.730 ± 0.001	14.050 ± 0.001	14.050 ± 0.001	87.070 ± 0.000
10	*Caryophyllaceae*	*Dianthus chinensis* L.	Diantus	Salad, tea	94.444 ± 0.922	80.793 ± 1.819	−3.137 ± 0.110	11.687 ± 2.201	12.106 ± 0.156	105.231± 2.077
11	*Caryophyllaceae*	*Gypsophila paniculata* L.	Veil	Infusion	88.312 ± 1.542	na	na	na	na	na
12	*Convolvulaceae*	*Convolvulus pseudoscammonia* C. Koch L.	Meadow bell	Infusion	89.878 ± 0.808	83.093 ± 4.473	−1.800 ± 0.171	5.767 ± 1.140	6.043 ± 0.120	107.501 ± 1.604
13	*Iridaceae*	*Gladiolus communis* L.	Gladiolus	Salad, garrison	79.592 ± 0.349	67.010 ± 0.001	0.730 ± 0.001	14.051 ± 0.008	14.069 ± 0.002	87.070 ± 0.001
14	*Lamiaceae*	*Mentha suaveolens* Ehrh.	Mentha suaveolens	Infusion	79.771 ± 0.902	na	na	na	na	na
15	*Magnoliaceae*	*Magnolia grandiflora* L.	Magnolia	Tea	82.151 ± 0.272	83.093 ± 4.473	−1.800 ± 0.171	5.767 ± 0.140	6.043 ± 0.410	107.501 ± 0.764
16	*Oleaceae*	*Jasminum sambac* (L.) Aiton	Jasmine of Arabia	Salad, tea	84.826 ± 0.104	83.987 ± 2.450	−2.927 ± 0.401	16.497 ± 0.901	16.757 ± 0.918	100.059 ± 0.129
17	*Orchidaceae*	*Phalaenopsis aphrodite* Rchb. f.	Orchid	na	98.172 ± 0.063	70.063 ± 1.670	10.030 ± 2.044	−7.103 ± 1.079	12.297 ± 2.201	325.161 ± 1.368
18	*Plumbaginaceae*	*Plumbago auriculata* Lam.	Celestine	Infusion	26.895 ± 0.872	54.340 ± 1.806	1.530 ± 0.041	−16.017 ± 0.154	16.208 ± 0.157	275.041 ± 0.289
19	*Rosaceae*	*Fragaria ×* Duchesne ex Rozier	Strawberry	na	94.215 ± 0.579	67.010 ± 0.001	0.730 ± 0.020	14.050 ± 0.001	14.069 ± 0.031	87.070 ± 0.011
20	*Rosaceae*	*Rosa hybrid* Vill.	Rose	Salad, desserts	79.739 ± 0.888	86.353 ± 0.915	0.030 ± 0.041	14.207 ± 0.059	14.207 ± 0.024	89.924 ± 0.005
21	*Solanaceae*	*Capsicum annuum* L.	Pepper	na	98.960 ± 0.921	na	na	na	na	na
22	*Solanaceae*	*Solanum laxum* Sprengel	False jasmine	na	85.197 ± 1.508	50.783 ± 2.100	11.903 ± 1.050	8.620 ± 0.219	14.798 ± 1.203	35.636 ± 0.832
23	*Verbenaceae*	*Aloysia citriodora* Palau	Cedrón	Tea	98.118 ± 0.847	na	na	na	na	na
24	*Verbenaceae*	*Lantana camara* L.	Lantana	Tea	86.986 ± 0.183	64.770 ± 0.143	2.703 ± 0.246	17.780 ± 1.362	17.991 ± 1.326	81.447 ± 1.244
Yellow flowers										
25	*Araceae*	*Aglaonema commutatum* Schott	Aglaonema	Non edible	91.958 ± 0.900	66.330 ± 1.411	−0.627 ± 0.055	28.877 ± 1.864	28.884 ± 0.186	91.205 ± 0.180
26	*Asteraceae*	*Anthemis tinctoria* L.	Golden Daisy	Colorant	78.726 ± 0.172	60.770 ± 2.463	21.140 ± 0.654	87.357 ± 2.326	89.878 ± 0.740	76.436 ± 0.139
27	*Asteraceae*	*Dahlia coccinea* Cav.	Dahlia	Salad	87.788 ± 0.184	61.363 ± 0.719	2.133 ± 0.201	66.667 ± 0.540	66.713 ± 0.281	88.181 ± 0.133
28	*Asteraceae*	*Dahlia pinnata* Cav.	Dahlia	Salad	74.519 ± 0.001	67.290 ± 0.646	16.523 ± 0.018	75.780 ± 0.546	77.561 ± 0.572	77.739 ± 0.060
29	*Brassicaceae*	*Diplotaxis tenuifolia* (L.) DC.	Rucula	Salad	87.218 ± 0.528	na	na	na	na	na
30	*Cannabaceae*	*Cannabis sativa* L.	Cannabis	Non edible	76.200± 0.914	na	na	na	na	na
31	*Celastraceae*	*Euonymus japonicus* Thunb.	Burning bush	Colorant	92.000 ± 1.028	na	na	na	na	na
32	*Fabaceae*	*Sophora japonica* L.	Acacia Japan	Infusion	73.629 ± 0.384	79.180 ± 1.871	−6.573 ± 0.116	22.023 ± 0.733	22.984 ± 0.107	106.592 ± 0.582
33	*Fabaceae*	*Senna papillosa* H.S. Irwin & Barneby	Senna	Non edible	73.823 ± 0.326	58.420 ± 4.265	11.060 ± 2.265	38.370 ± 4.099	40.000 ± 5.911	74.380 ± 4.294
34	*Juglandaceae*	*Pterocarya stenoptera* C. DC.	Chinese fresno	Non edible	84.572 ± 0.611	73.000 ± 1.279	−5.970 ± 0.214	32.910 ± 1.255	33.455 ± 1.245	100.342 ± 0.283
35	*Lamiaceae*	*Ocimum basilicum* L.	Basil	Salad, tea	94.680 ± 1.525	80.893 ± 3.409	−7.227 ± 0.657	32.187 ± 1.419	32.990 ± 0.712	102.602 ± 0.691
36	*Malvaceae*	*Gossypium arboreum* L.	Cotton	Non edible	81.410 ± 0.114	80.873 ± 3.412	−7.243 ± 0.713	32.201 ± 1.403	33.120 ± 0.725	102.612 ± 0.743
37	*Plantaginaceae*	*Plantago major* L.	Plantain	Infusion	85.714 ± 0.200	na	na	na	na	na
38	*Polygonaceae*	*Fallopia aubertii* (L.Henry) Holub	Gabriela falloppio	na	92.411 ± 0.332	na	na	na	na	na
39	*Portulacaceae*	*Portulaca oleracea* L.	Purslane	Salad	98.092 ± 0.661	71.643 ± 0.965	4.887 ± 0.240	29.533 ± 0.397	29.906 ± 0.401	80.624 ± 0.311
40	*Rubiaceae*	*Gardenia jasminoides* J. Ellis	Gardenia	Colorant	82.213 ± 0.283	84.253 ± 3.833	3.453 ± 0.116	46.923 ± 0.470	47.050 ± 0.161	85.833 ± 0.179
41	*Solanaceae*	*Solanum lycopersicum* L.	Tomato	na	87.255 ± 0.261	77.200 ± 1.001	−4.500 ± 0.121	22.034 ± 0.704	23.036 ± 0.111	106.603 ± 0.612
42	*Verbenaceae*	*Lantana camara* L.	Lantana	Tea	88.122 ± 0.706	50.570 ± 0.875	18.403 ± 0.431	48.440 ± 0.128	51.823 ± 0.135	69.101 ± 1.018
Orange flowers										
43	*Acanthaceae*	*Justicia aurea* Schltdl.		na	94.207 ± 0.184	68.817 ± 1.399	4.123 ± 0.456	88.640 ± 3.326	88.736 ± 3.433	87.386 ± 0.191
44	*Bignoniaceae*	*Tecoma capensis* (Thunb.) Lindl.	Cape honeysuckle	Infusion	72.225 ± 0.506	53.233 ± 2.266	40.633 ± 2.053	34.833 ± 2.048	53.572 ± 0.299	40.635 ± 3.084
45	*Gesneriaceae*	*Drymonia affinis* (Mansf.) Wiehler	Drymonia	na	73.209 ± 0.172	49.510 ± 1.247	24.983 ± 0.582	32.520 ± 0.963	41.010 ± 1.063	52.490 ± 0.519
46	*Gesneriaceae*	*Drymonia brochidodroma* Wiehler	Drymonia	na	91.041 ± 0.703	46.622 ± 1.207	25.043 ± 0.604	32.511 ± 1.000	41.002 ± 1.333	52.512 ± 1.302
47	*Lythraceae*	*Punica granatum* L.	Pomegranate	Infusion	69.105 ± 0.437	43.093 ± 2.765	47.340 ± 0.356	36.738 ± 0.217	59.743 ± 0.133	38.153 ± 0.375
48	*Zigniberaceae*	*Renealmia alpinia* (Rottb.) Maas	Honeyy bract	Spice	89.126 ± 1.333	52.111 ± 0.224	42.175 ± 0.126	35.101 ± 0.243	54.872 ± 0.302	39.825 ± 2.126

na, not available.

**Table 2:** Mean color parameter values, humidity, and culinary uses (according to Coyago et al., (2017) [32] and The-Plant-List, (2019) [33]) of red and pink flowers.

Samples	Family	Species	Common Name	Culinary Use	Humidity (%)	L*	a*	b*	C*_ab_	h_ab_
Red flowers										
49	*Acanthaceae*	*Aphelandra squarrosa* Nees	Zebra plant	na	73.202 ± 3.184	30.847 ± 1.259	49.437 ± 0.942	32.257 ± 0.541	59.030 ± 1.028	33.142 ± 0.339
50	*Amaranthaceae*	*Celosia argentea* L.	Cockscomb	na	71.704 ± 0.126	36.837 ± 2.878	23.600 ± 2.946	−2.833 ± 0.342	23.817 ± 2.999	352.621 ± 0.629
51	*Apocynaceae*	*Catharanthus roseus* (L.) G. Don	Vinca rosea	Non-edible	96.519 ± 0.015	46.001 ± 0.012	49.863 ± 0.285	−7.123 ± 0.081	50.370 ± 0.270	351.865 ± 0.137
52	*Apocynaceae*	*Nerium oleander* L.	Flower laurel	Non-edible	89.739 ± 3.621	67.077 ± 1.693	19.380 ± 1.427	3.250 ± 0.658	19.810 ± 1.583	10.429 ± 1.409
53	*Araceae*	*Anthuriumandraeanum* Linden ex	Anus	Non-edible	94.096 ± 1.525	50.630 ± 0.404	32.917 ± 1.165	11.283 ± 1.585	34.814 ± 1.226	18.895 ± 2.255
54	*Balsaminaceae*	*Impatiens balsamina* L.	Joy of home	Salad, desserts	91.049 ± 0.211	41.543 ± 2.466	47.893 ± 4.686	23.887 ± 3.367	53.528 ± 4.758	26.449 ± 1.238
55	*Balsaminaceae*	*Impatiens walleriana* Hook. F.	My dear	Salad, desserts	85.202 ± 0.984	50.287 ± 2.228	57.220 ± 2.573	19.490 ± 2.556	60.463 ± 2.626	18.770 ± 1.556
56	*Begoniaceae*	*Begonia cavaleriei* H. Lév.	Begonia	na	76.748 ± 0.253	54.793 ± 4.344	19.460 ± 2.899	12.267 ± 0.498	23.311 ± 2.381	33.935 ± 3.613
57	*Begoniaceae*	*Begonia cucullata* Willd.	Sugar flower	Salad, desserts	59.290 ± 0.127	37.443 ± 0.873	36.870 ± 2.315	15.157 ± 2.171	39.931 ± 2.942	21.995 ± 1.802
58	*Begoniaceae*	*Begonia × tuberhybrida* Voss	Begonia	Salad, desserts	99.736 ± 0.834	32.067 ± 1.391	53.383 ± 2.036	33.483 ± 1.014	63.018 ± 2.134	32.121 ± 0.720
59	*Caryophyllaceae*	*Dianthus caryophyllus* L.	Carmination	Fruit salad	95.775 ± 0.106	39.863 ± 4.495	50.847 ± 3.674	21.403 ± 3.633	55.206 ± 3.751	22.762 ± 2.616
60	*Ericaceae*	*Rhododendron simsii* Planch.	Azalea	Non-edible	41.527 ± 2.572	41.543 ± 2.466	47.893 ± 3.468	23.887 ± 3.367	53.528 ± 1.265	26.449 ± 1.238
61	*Escalloniaceae*	*Escallonia rubra* Pers.	Escalloniacea	na	82.780 ± 0.427	na	na	na	na	na
62	*Euphorbiaceae*	*Euphorbia milii* Des Moul.	Crown of christ	Non-edible	98.893 ± 0.165	40.683 ± 2.678	32.693 ± 2.356	13.493 ± 1.152	35.384 ± 1.765	22.483 ± 2.048
63	*Fabaceae*	*Brownea macrophylla* Linden	Panama flame tree	na	87.209 ± 0.217	na	na	na	na	na
64	*Geraniaceae*	*Pelargonium peltatum* (L.) L´Hér.	Gitanilla	na	96.390 ± 0.028	14.157 ± 0.337	17.037 ± 0.491	1.823 ± 0.161	17.135 ± 0.472	6.124 ± 0.694
65	*Geraniaceae*	*Pelargonium x hortorum* H. Bailey	Geranium	Salad, desserts	86.548 ± 0.281	30.353 ± 2.168	46.593 ± 2.431	27.520 ± 2.095	54.119 ± 2.408	30.563 ± 0.945
66	*Lamiaceae*	*Salvia splendens* Sellow ex Schult.	Red sage	Garrison	72.018 ± 0.164	38.813 ± 2.189	24.673 ± 1.401	21.833 ± 0.132	33.424 ± 1.121	42.183 ± 1.443
67	*Malvaceae*	*Malvaviscus arboreus* Cav.	Marshmallow	Infusion	86.152 ± 0.909	40.990 ± 1.676	47.580 ± 2.984	25.150 ± 3.289	53.833 ± 3.015	27.789 ± 1.699
68	*Onagraceae*	*Fuchsia magellanica* Lam.	Fuchsia	Tea	81.264 ± 0.009	44.210 ± 2.187	34.773 ± 0.917	3.037 ± 0.703	34.980 ± 1.053	5.963 ± 0.856
69	*Rosaceae*	*Rosa hybrid* Vill.	Rose	Salad, desserts	78.682 ± 0.325	28.440 ± 1.591	46.303 ± 0.280	17.863 ± 1.013	49.635 ± 0.356	21.100 ± 1.027
70	*Papaveraceae*	*Papaver rhoeas* L.	Wheat poppy	Garrison	72.111 ± 0.263	40.683 ± 2.678	32.693 ± 3.566	13.493 ± 1.520	35.384 ± 3.653	22.483 ± 2.048
71	*Rubiaceae*	*Warszewiczia coccinea* Klotzsch	Chaconia	Tea	83.000 ± 1.381	34.253 ± 1.363	46.237 ± 2.908	12.033 ± 0.526	47.777 ± 2.944	14.607 ± 0.316
72	*Scrophulariaceae*	*Antirrhinum majus* L.	Dragon mouth	Salad	94.967 ± 0.288	24.040 ± 1.152	18.920 ± 0.593	15.050 ± 1.283	24.201 ± 0.367	38.489 ± 3.222
73	*Scrophulariaceae*	*Russelia equisetiformis* Schltdl. &	Ruselia	Infusion	89.257 ± 0.129	41.543 ± 2.466	47.893 ± 4.686	23.887 ± 3.367	53.528 ± 4.765	26.449 ± 1.238
74	*Solanaceae*	*Petunia hybrida* Vilm.	Petunia	Salad, desserts	90.986 ± 5.425	34.253 ± 1.363	46.237 ± 2.908	12.033 ± 0.526	47.777 ± 2.944	14.607 ± 0.316
75	*Verbenaceae*	*Lantana camara* L.	Lantana	Tea	83.774 ± 2.182	33.210 ± 0.439	38.610 ± 2.384	30.187 ± 0.335	49.124 ± 0.282	38.024 ± 0.478
76	*Verbenaceae*	*Verbena × hybrid* Groenland	Verbena	Salad, garrison	85.436 ± 0.023	26.913 ± 0.674	36.787 ± 3.852	14.537 ± 0.872	39.560 ± 3.868	21.641 ± 1.176
Pink flowers										
77	*Amaranthaceae*	*Celosia argentea* L.	Cockscomb	na	72.421 ± 0.184	19.443 ± 2.286	34.023 ± 1.479	4.120 ± 1.085	34.286 ± 1.538	6.948 ± 2.004
78	*Apocynaceae*	*Nerium oleander* L.	Flower laurel	Non-edible	82.058 ± 0.327	58.847 ± 3.279	29.247 ± 4.101	−2.743 ± 0.145	29.387 ± 4.139	354.364 ± 0.711
79	*Begoniaceae*	*Begonia argentea* Linden	Begonia	na	91.547 ± 1.522	na	na	na	na	na
80	*Bromeliaceae*	*Guzmania hybrid*	Guzmania	na	89.651 ± 0.001	46.660 ± 0.233	15.770 ± 0.885	9.340 ± 0.524	18.343 ± 0.488	30.703 ± 2.838
81	*Caryophyllaceae*	*Dianthus caryophyllus* L.	Carmination	Fruit salad	84.678 ± 0.325	43.320 ± 1.501	34.690 ± 0.436	3.987 ± 0.127	34.918 ± 0.447	6.558 ± 0.126
82	*Caryophyllaceae*	*Saponaria officinalis* L.	Soap flower	Non-edible	81.110 ± 0.202	68.560 ± 0.291	3.883 ± 0.946	−3.333 ± 0.818	5.133 ± 0.760	318.436 ± 5.235
83	*Ericaceae*	*Rhododendron simsii* Planch.	Azalea indica	Non-edible	74.579 ± 0.299	82.190 ± 1.669	5.943 ± 0.421	8.860 ± 0.739	10.686 ± 0.396	56.081 ± 4.033
84	*Fabaceae*	*Trifolium cernuum* Brot.	Four leaf clover	Salad, tea	81.411 ± 0.303	50.783 ± 1.421	11.903 ± 0.850	8.620 ± 1.397	14.798 ± 0.203	35.636 ± 5.324
85	*Gentianaceae*	*Eustoma grandiflorum* G. Don	Eustoma	na	92.072 ± 0.099	66.407 ± 4.493	4.067 ± 0.668	5.233 ± 0.486	6.630 ± 0.792	52.333 ± 2.000
86	*Geraniaceae*	*Pelargonium domesticum* Bailey	Real geranium	Salad, desserts	85.698 ± 0.785	15.783 ± 1.532	16.793 ± 1.561	1.757 ± 0.875	17.147 ± 1.675	12.053 ± 1.4800
87	*Geraniaceae*	*Pelargonium × hortorum* Bailey	Geranium	Salad, desserts	75.523 ± 0.185	52.563 ± 0.198	29.107 ± 0.233	2.177 ± 0.437	29.200 ± 0.234	4.016 ± 0.172
88	*Hydrangeaceae*	*Hydrangea petiolaris S.* & Zucc.	Hydrangea	Infusion	74.361 ± 0.725	58.440 ± 1.439	24.807 ± 1.436	−3.767 ± 0.372	25.092 ± 1.463	351.369 ± 0.558
89	*Lythraceae*	*Cuphea hyssopifolia* Kunth	False breccia	Infusion	95.948 ± 0.811	na	na	na	na	na
90	*Lythraceae*	*Lagerstroemia indica* L.	Jupiter tree	Tea	87.561 ± 0.347	47.777 ± 0.128	27.050 ± 0.622	−4.687 ± 0.063	27.456 ± 0.622	350.173 ± 0.302
91	*Malvaceae*	*Gossypium arboreum* L.	Cotton	Non-edible	82.733 ± 5.421	51.887 ± 5.484	10.090 ± 0.617	9.913 ± 0.588	14.162 ± 0.078	44.522 ± 3.431
92	*Nyctaginaceae*	*Mirabilis jalapa* L.	Night Dondiego	Colorant	85.593 ± 0.206	53.100 ± 1.815	15.547 ± 0.213	7.133 ± 0.445	17.175 ± 0.143	24.759 ± 1.630
93	*Orchidaceae*	*Phalaenopsis aphrodite* Rchb. F.	Orchid	na	92.271 ± 0.358	50.783 ± 2.100	11.903 ± 0.805	8.620 ± 1.321	14.798 ± 0.120	35.636 ± 3.024
94	*Portulacaceae*	*Portulaca oleracea* L.	Purslane	Salad	85.529 ± 3.674	35.780 ± 0.344	18.983 ± 0.144	27.170 ± 0.265	33.145 ± 0.136	55.085 ± 0.466
95	*Rosaceae*	*Rosa hybrid*	Rose	Salad, desserts	89.769 ± 0.105	47.763 ± 3.951	55.923 ± 5.390	19.007 ± 0.679	59.088 ± 5.045	18.886 ± 1.936
96	*Verbenaceae*	*Verbena × hybrid* G. & Rümpler	Verbena	Salad, garrison	84.040 ± 0.037	41.467 ± 3.153	19.337 ± 0.140	2.330 ± 0.437	19.486 ± 0.153	6.793 ± 1.117

na, not available.

**Table 3:** Mean color parameter values, humidity, and culinary uses (according to Coyago et al., (2017) [32] and The-Plant-List, (2019) [33]) of lilac and blue flowers.

Samples	Family	Species	Common name	Culinary use	Humidity (%)	L*	a*	b*	C*_ab_	h_ab_
Lilac flowers										
97	*Amaryllidaceae*	*Allium schoenoprasum* L.	Chives	Salad, garrison	74.820 ± 0.222	20.333 ± 3.502	2.915 ± 0.126	−0.147 ± 0.001	2.983 ± 0.119	359.606 ± 0.012
98	*Apocynaceae*	*Catharanthus roseus* L.	Vinca rosea	Non-edible	87.648 ± 1.291	54.233 ± 2.573	30.217 ± 2.145	−18.707 ± 1.692	35.553 ± 2.139	328.223 ± 1.971
99	*Asteraceae*	*Centaurea seridis* L.	Spiny broom	na	93.516 ± 0.883	na	na	na	na	na
100	*Asteraceae*	*Cichorium intybus* L.	Chicory of Brussels	Salad, tea	98.378 ± 0.283	na	na	na	na	na
101	*Asteraceae*	*Osteospermun fruticosum* Norl.	Cape margarita	Tea	82.523 ± 0.317	47.673 ± 2.091	29.153 ± 0.459	−15.303 ± 0.110	32.927 ± 0.379	332.286 ± 0.488
102	*Brassicaceae*	*Alyssum montanum* L.	Garlic herb	Infusion	81.159 ± 0.395	na	na	na	na	na
103	*Campanulaceae*	*Campanula carpatica* Jacq.	Little bell	na	85.496 ± 0.152	na	na	na	na	na
104	*Geraniaceae*	*Pelargonium domesticum* Bailey	Real geranium	Salad, desserts	92.894 ± 0.800	58.660 ± 4.038	25.157 ± 1.518	−12.213 ± 0.319	31.101 ± 1.325	335.566 ± 1.634
105	*Geraniaceae*	*Pelargonium × hortorum* Bailey	Common geranium	Salad, desserts	83.186 ± 0.001	38.290 ± 2.736	53.403 ± 1.538	−11.497 ± 0.678	54.633 ± 1.355	167.827 ± 1.051
106	*Lamiaceae*	*Mentha ×piperita* L.	Peppermint	Salad, garrison	94.503 ± 0.063	na	na	na	na	na
107	*Lamiaceae*	*Ocimum basilicum* L.	Basil	Salad, tea	89.677 ± 1.222	41.423 ± 1.120	6.436 ± 0.311	−4.879 ± 0.921	8.124 ± 0.343	322.701 ± 4.206
108	*Malvaceae*	*Hibiscus syriacus* L.	Rose of Syria	Salad, tea	70.778 ± 0.747	53.743 ± 0.282	18.460 ± 1.136	−12.697 ± 0.616	22.407 ± 1.202	325.356 ± 0.302
109	*Nyctaginaceae*	*Bougainvillea spectabilis* Willd.	Bougainvillea	Infusion	86.967 ± 1.558	50.753 ± 0.741	6.963 ± 1.002	1.790 ± 0.310	7.309 ± 0.911	16.291 ± 3.413
110	*Plumbaginaceae*	*Limonium sinuatum* (L.) Miller	Always alive	Additive	91.656 ± 0.184	na	na	na	na	na
111	*Polygonaceae*	*Fallopia aubertii* (L. Henry) Holub	Gabriela falloppio	na	99.350 ± 1.200	na	na	na	na	na
112	*Solanaceae*	*Petunia hybrida* Vilm.	Petunia	Salad, desserts	86.068 ± 0.315	69.300 ± 0.566	9.533 ± 1.230	−4.087 ± 1.287	10.390 ± 0.346	337.496 ± 1.963
113	*Solanaceae*	*Solanum rantonnetti* Carrière	Blue flower solano	Infusion	83.018 ± 0.126	na	na	na	na	na
114	*Verbenaceae*	*Verbena × hybrid* G. & Rümpler	Verbena	Salad, garrison	84.238 ± 1.282	41.367 ± 1.086	6.397 ± 0.303	−4.913 ± 0.949	8.081 ± 0.379	322.707 ± 4.156
115	*Verbenaceae*	*Vitex agnus-castus* L.	Chilli pepper	Infusion	82.257 ± 0.666	33.417 ± 1.246	9.887 ± 1.320	−14.997 ± 2.539	17.967 ± 2.844	303.628 ± 1.419
Blue flowers										
116	*Amaryllidaceae*	*Agapanthus africanus* Hoffmanns	African lily	Infusion	82.523 ± 5.401	57.173 ± 2.173	4.543 ± 0.162	−15.723 ± 1.030	16.367 ± 0.233	286.099 ± 0.508
117	*Convolvulaceae*	*Convolvulus althaeoides* L.	Bell of the virgin	Non-edible	89.100 ± 0.172	61.887 ± 1.980	12.333 ± 1.512	−3.312 ± 0.001	12.709 ± 1.520	345.099 ± 1.657
118	*Gesneriaceae*	*Saintpaulia ionantha* Wendland	African violet	Salad	89.577 ± 0.065	na	na	na	na	na
119	*Goodeniaceae*	*Scaevola aemula* R. Bronw	Flower fan	Infusion	93.812 ± 0.273	36.913 ± 1.770	8.497 ± 0.619	0.539 ± 0.016	8.431 ± 0.623	3.380 ± 0.521
120	*Lamiaceae*	*Agastachefoeniculum* Kuntze	Anise hyssop	Salad, desserts	79.731 ± 0.288	na	na	na	na	na
121	*Lamiaceae*	*Lavandula angustifolia* Mill.	Lavender	Infusion	88.511 ± 0.173	37.582 ± 1.607	8.8977 ± 0.639	−12.101 ± 0.812	15.026 ± 0.922	306.212 ± 0.144
122	*Lamiaceae*	*Rosmarinus officinalis* L.	Rosemary	Garrison, desserts	89.483 ± 0.211	na	na	na	na	na
123	*Passiofloraceae*	*Passiflora × belotti*	Flower of the passion	Tea	97.826 ± 0.742	na	na	na	na	na
124	*Polygonaceae*	*Polygala vulgaris* L.	Common sparrow	Infusion	88.391 ± 0.364	49.780 ± 1.113	17.963 ± 1.319	−10.847 ± 2.043	21.006 ± 1.320	329.058 ± 3.214
125	*Solanaceae*	*Petunia × hybrida* Vilm.	Petunia	Salad, desserts	83.425 ± 0.779	23.210 ± 1.458	23.027 ± 1.216	−25.717 ± 1.197	34.519 ± 1.703	311.811 ± 0.177

na, not available.

**Table 4:** Carotenoid contents (μg/g dry weight) of white, yellow, and orange flowers and retinol activity equivalents.

Species		Phytoene	Lutein Epoxide	Luteoxanthin	Antheraxanthin	9-*Cis*-Violaxanthin	Violaxanthin	Lutein	9-*Cis*-Anteraxanthin	Zeinoxanthin	β-Carotene	α-Carotene	TOTAL	Retinol Activity Equivalents FW
White flowers														
1	*S. montanum*						47.724 ± 0.633	96.839 ± 1.304			32.235 ± 2.525	82.001 ± 0.789	258.863 ± 0.843	0.629 ± 0.123
2	*C. comosum*							14.816 ± 0.003			79.101 ± 2.045		93.917 ± 1.700	0.059 ± 0.018
3	*A. africanus*				3.648 ± 0.460			4.478 ± 0.373					8.125 ± 0.069	
4	*C. sativum*							141.747 ± 0.501	22.734 ± 2.594		103.121 ±1.176		267.601 ± 1.238	0.352 ± 0.023
5	*N. oleander*							2.748 ± 0.3122					2.748 ± 0.3122	
6	*T.jasminoides*							3.581 ± 0.527					3.581 ± 0.527	
7	*H. arborescens*							26.995 ± 2.084			3.745 ± 0.251		30.740 ± 0.195	0.044 ± 0.015
8	*M. incana*							2.523 ± 0.411					2.523 ± 0.411	
9	*C. shetleri*				11.357 ± 0.434			11.675 ± 0.446					23.033 ± 0.068	
10	*D. chinensis*												nd	
11	*G. paniculata*				6.649 ± 0.855			9.124 ± 0.930			18.801 ± 0.232		33.854 ± 0.3168	0.980 ± 0.029
12	*C. scammonia*		5.989 ± 0.770					5.929 ± 0.235					11.918 ± 0.077	
13	*G. communis*							3.146 ± 0.107					3.146 ± 0.107	
14	*M. suaveolens*		38.523 ± 2.621	6.901 ± 0.415			21.677 ± 1.936	71.278 ± 0.439			11.611 ± 0.001		149.990 ± 0.687	0.195 ± 0.001
15	*M. grandiflora*	20.359 ± 1.259											20.359 ± 1.259	
16	*J. sambac*							4.877 ± 0.136					4.877 ± 0.136	
17	*P. aphrodite*							1.901 ± 0.411			6.822 ± 0.330		8.273 ± 0.125	0.063 ± 0.023
18	*P. auriculata*												nd	
19	*F. ×* *ananassa*												nd	
20	*Rosa hybrid*	16.801 ± 0.001			6.418 ± 0.546			9.970 ± 0.702					33.249 ± 0.153	
21	*C. annuum*		20.435 ± 0.642		18.313 ± 0.576		16.199 ±0.509	76.460 ± 0.468			8.272 ± 0.161		139.680 ± 0.618	0.141 ± 0.066
22	*S. laxum*		10.427 ± 0.360		3.734 ± 0.101			4.730 ± 0.163			6.682 ± 0.944		25.572 ± 0.845	0.028 ± 0.015
23	*A. citriodora*							1.741 ± 0.264					1.741 ± 0.264	
24	L. camara*	44.352 ± 1.586									6.914 ± 0.238	13.515 ± 0.321	64.782 ± 0.420	0.148 ± 0.011
		*13-*Cis*-violaxanthin (43.503 ± 0.722); 9-*Cis*-lutein (140.712 ± 0.586); Zeaxanthin (147.8 ± 2.502); β-Cryptoxanthin (361.422 ± 7.638); α-*Cis*-anteraxanthin (232.215 ± 12.126).												
Yellow flowers														
25	*A. commutatu*		10.424 ± 0.162				13.040 ± 0.203	43.188 ± 0.462			12.028 ± 0.245		78.680 ± 0.402	0.085 ± 0.024
26	*A. tinctoria*		9.338 ± 0.574				10.240 ± 0.630						19.578 ± 0.093	
27	*D. coccinea*												nd	
28	*D. pinnata*							19.521 ± 0.613			20.492 ± 0.644		40.013 ± 0.097	0.208 ± 0.044
29	*D. tenuifolia*		17.464 ± 0.549				33.122 ± 3.642		41.066 ± 1.291		13.755 ± 0.432		105.408 ± 0.455	0.021 ± 0.021
30	*C. sativa*							16.949 ± 0.585			2.878 ± 0.099		19.826 ± 0.057	0.057 ± 0.035
31	*E. japonicus*							31.441 ± 0.946					31.441 ± 0.946	
32	*S. japonica*							38.878 ± 1.341			100.347 ± 3.461		139.225 ± 0.369	2.208 ± 0.010
33	*S. papillosa*						86.001 ± 0.021	1204.010 ± 0.062		1311.876 ± 0.052	170.316 ± 0.001		2772.202 ± 0.056	3.477 ± 0.027
34	*P. stenoptera*		3.623 ± 0.803	1.130 ± 0.226			3.991 ± 0.593	23.966 ± 1.709					32.709 ± 0.278	
35	*O. basilicum*		7.293 ± 0.106				7.992 ± 0.124	205.176 ± 1.318			284.137 ± 1.924		504.597 ± 2.895	1.255 ± 0.030
36	*G. arboreum*							5.150 ± 0.362					5.150 ± 0.362	
37	*P. major*						31.395 ± 0.489	136.768 ± 3.277					168.162 ± 0.314	
38	*F. aubertii*							3.725 ± 0.394					3.725 ± 0.394	
39	*P. oleracea*							334.85 ± 8.972	433.409 ± 0.182	239.780 ± 3.720	4.357 ± 0.685		1012.431 ± 7.860	0.051 ± 0.041
40	*G. jasminoides*	6.895 ± 0.150											6.895 ± 0.150	
41	*S. lycopersicum*		31.885 ± 0.609				20.207 ± 1.752	35.960 ± 1.748					88.052 ± 0.738	
42	*L. camara^a^*	12.744 ± 0.648	75.829 ± 2.937			92.465 ± 0.823	43.500 ± 0.307	63.792 ± 3.421	54.906 ± 2.379		50.828 ± 0.461	731.514 ± 7.631	2056.065 ± 7.148	4.796 ± 1.027
Orange flowers														
43	*J. aurea*						47.880 ± 0.071						47.880 ± 0.071	
44	*T. capensis*		6.595 ± 0.751		1.844 ± 0.260			6.320 ± 0.406	6.621 ± 0.426				37.646 ± 0.241	0.188 ± 0.015
45	*D. affinis*					22.283 ± 0.003	30.403 ± 0.013	43.261 ± 0.001			16.277 ± 0.034		112.225 ± 0.026	0.364 ± 0.007
46	*D. brochidodroma*					97.582 ± 0.011	258.828 ± 0.078	76.733 ± 0.022					433.144 ± 0.080	
47	*P. granatum*							24.981 ± 0.309			8.891 ± 0.279		33.872 ± 0.260	0.229 ± 0.075
48	*R. alpinia*									220.643 ± 0.06	1372.181 ± 0.001	1451.916 ± 0.003	3044.739 ± 2.120	19.058 ± 0.019

nd, not detectable.

**Table 5:** Carotenoid contents (μg/g dry weight) of red and pink flowers and retinol activity equivalents.

	Species	Reaction	Phytoene	Lutein Epoxide	Antheraxanthin	9-*Cis*-Violaxanthin	Violaxanthin	Lutein	9-*Cis*-Anteraxanthin	Zeinoxanthin	β-Cryptoxanthin	β-Carotene	α-Carotene	Total	Others Carotenoids	Retinol Activity Equivalents FW
Red flowers																
49	*A. squarrosa*	S					140.001 ± 0.001	208.963 ± 0.012		32.298 ± 0.001				381.262 ± 0.003		
50	*C. argentea*	S				2.518 ± 0.556		8.205 ± 0.637	8.452 ± 0.893			2.900 ± 0.286		22.074 ± 0.151		0.068 ± 0.084
51	*C. roseus*							3.691 ± 0.721						3.691 ± 0.721		
52	*N.oleander*							3.431 ± 0.439						3.431 ± 0.439		
53	*A. andraeanum*							11.101 ± 0.109				3.041 ± 0.490		14.142 ± 0.122		0.015 ± 0.070
54	*I. balsamina*							2.890 ± 0.209						2.890 ± 0.209		
55	*I. walleriana*							2.908 ± 0.196						2.908 ± 0.196		
56	*B. cavaleriei*	S										3.689 ± 0.119		21.277 ± 0.092	Lycopene (17.588 ± 0.567)	0.072 ± 0.042
57	*B. andraeanum*	S	3.950 ± 0.114											3.950 ± 0.114		
58	*B. × tuberhybrida*	S						8.719 ± 0.910						8.719 ± 0.910		
59	*D. caryophyllus*							15.862 ± 0.408						15.862 ± 0.408		
60	*R. simsii*	S						79.036 ± 0.063						79.036 ± 0.063		
61	*E. rubra*							5.163 ± 0.224						5.163 ± 0.224		
62	*E. milii*							7.357 ± 0.103						7.357 ± 0.103		
63	*B. macrophylla*	S								17.165 ± 0.023	33.391 ± 0.072	203.433 ± 0.001		377.425 ± 0.009	Luteoxanthin (98.725 ± 0.001); 9-*Cis*-β-cryptoxanthin (24.704 ± 0.011)	2.385 ± 0.001
64	*P. peltatum*							3.280 ± 0.108						3.280 ± 0.108		
65	*P. × hortorum*							3.521 ± 0.491						3.521 ± 0.491		
66	*S. splendens*	S						5.548 ± 0.234						5.548 ± 0.234		
67	*M. arboreus*	S						140.300 ± 0.004	7.074 ± 0.295					147.374 ± 0.023		
68	*F. magellanica*				5.269 ± 0.537			6.497 ± 0.001						11.766 ± 0.045		
69	*Rosa hybrid*											6.800 ± 0.303		6.800 ± 0.303		0.122 ± 0.016
70	*P. rhoeas*	S						66.753 ± 0.823						66.753 ± 0.823		
71	*W. coccinea*	S						97.236 ± 0.001						97.236 ± 0.001		
72	*A. majus*													nd		
73	*R. equisetiformis*	S		9.144 ± 0.732				140.300 ± 0.001			4.252 ± 0.340	4.085 ± 0.106		161.976 ± 0.164	Neochrome (4.252 ± 0.340)	0.055 ± 0.001
74	*Petunia × hybrid*							6.019 ± 0.111						6.019 ± 0.111		
75	*L. camara*		33.946 ± 1.469			25.415 ± 2.768	46.326 ± 1.267	36.794 ± 0.959	25.694 ± 0.315		55.198 ± 1.267	1.542 ± 0.460	5.587 ± 0.287	304.721 ± 0.183		0.021 ± 0.000
76	*Verbena × hybrid*					30.303 ± 0.525	11.906 ± 1.700	58.361 ± 0.004	9.303 ± 0.002			26.972 ± 0.011		106.289 ± 0.017	15-*Cis*-violaxanthin (11.711 ± 1.600); 9-*Cis*-lutein (32.001 ±1.229)	0.328 ± 0.001
Pink flowers																
77	*C. argentea*						15.714 ± 0.421	100.672± 2.836						116.324± 0.303		
78	*N. oleander*							0.972 ± 0.003						0.972 ± 0.003		
79	*B. argentea*	S						182.793± 0.734				43.165 ± 0.181	15.310 ± 0.194	241.268± 0.594		0.803 ± 0.003
80	*Guzmania hybrid*		126.450 ± 0.852	2.912 ± 0.129				7.763 ± 0.107				13.867 ± 0.782		150.992± 0.859		0.119 ± 0.006
81	*D. caryophyllus*							1.662 ± 0.004						1.662 ± 0.004		
82	*S. officinalis*							9.544 ± 0.984						9.544 ± 0.984		
83	*R.simsii*							0.718 ± 0.074						0.718 ± 0.074		
84	*T. cernuum*		2.776 ± 0.304					2.041 ± 0.022						4.817 ± 0.041		
85	*E. grandiflorum*							0.966 ± 0.039						0.966 ± 0.039		
86	*P. domesticum*							2.078 ± 0.120						2.078 ± 0.120		
87	*P. × hortorum*													nd		
88	*H. petiolaris*							7.3 ± 0.2						7.3 ± 0.0		
89	*C. hyssopifolia*			19.298 ± 1.331				60.944 ± 1.834	11.306 ± 0.780			61.105 ± 0.421		157.029 ± 0.638	Luteoxanthin (4.375 ± 0.132)	0.209 ± 0.004
90	*L. indica*							4.385 ± 0.308				8.476 ± 0.595		12.861 ± 0.069		0.088 ± 0.000
91	*G. arboreum*							0.700 ± 0.001						0.700 ± 0.001		
92	*M. jalapa*	S						4.147 ± 0.328						4.147 ± 0.328		
93	*P. aphrodite*			6.858 ± 0.510				8.376 ± 0.802	8.742 ± 0.837			20.361 ± 1.252		44.338 ± 0.284		0.131 ± 0.002
94	*P. oleracea*							156.999 ± 3.163	355.241 ± 3.700	120.763 ± 5.221				632.974 ± 6.136		
95	*Rosa hybrid*		6.718 ± 0.846		9.657 ± 0.102			14.962 ± 0.188	5.379 ± 0.678			27.610 ± 0.205		64.238 ± 0.531		0.235 ± 0.002
96	*Verbena × hybrid*							10.142 ± 1.038				2.544 ± 0.048		12.685 ± 0.127		0.034 ± 0.001

S, saponified; nd, not detectable.

**Table 6:** Carotenoid contents (μg/g dry weight) of lilac and blue flowers and retinol activity equivalents.

	Species	Reaction	Phytoene	Lutein Epoxide	Luteoxanthin	Antheraxanthin	9-*Cis*-Violaxanthin	Violaxanthin	Lutein	9-*Cis*-Anteraxanthin	Zeinoxanthin	β-Cryptoxanthin	β-Carotene	α-Carotene	Total	Retinol Activity Equivalents FW
Lilac flowers																
97	*A. schoenoprasum*						14.146 ± 1.277	10.807 ± 0.976	36.072± 3.256				9.041 ± 0.081		70.066 ± 0.421	0.190 ± 0.001
98	*C. roseus*	S													nd	
99	*C. seridis*														nd	
100	*C. intybus*								2.096 ± 0.105						2.096 ± 0.105	
101	*O. fruticosum*								3.892 ± 0.815						3.892 ± 0.815	
102	*A. montanum*	S						10.610 ± 0.974	45.785± 0.420				24.954 ± 0.222		81.349 ± 0.575	0.100 ± 0.001
103	*C. carpatica*					6.957 ± 0.012			8.320 ± 0.001				20.323 ± 0.007		35.600 ± 0.064	0.020 ± 0.000
104	*Pelargonium × domesticum*								0.739 ± 0.071						0.739 ± 0.071	
105	*Pelargonium × hortorum*	S													nd	
106	*Mentha × piperita*	S		23.427 ± 0.415	5.907 ± 0.113			9.493 ± 0.182	70.580± 0.664				38.600 ± 0.141		148.008 ± 0.943	0.177 ± 0.002
107	*O. basilicum*			16.781 ± 1.022	5.105 ± 1.026			7.611 ± 0.100	37.663 ± 2.341				22.492 ± 0.864		88.873 ± 0.611	0.294 ± 0.007
108	*H. syriacus*	S							3.869 ± 0.481						3.869 ± 0.481	
109	*B. spectabili*	S						12.183 ± 1.010	33.282 ± 0.820						45.465 ± 0.141	
110	*L. sinuatum*												3.231 ± 0.152		3.231 ± 0.0152	0.240 ± 0.004
111	*F. aubertii*								5.407 ± 0.107						5.407 ± 0.107	
112	*Petunia × hybrida*								9.113 ± 0.126						9.113 ± 0.126	
113	*S. rantonnetti*		8.307 ± 0.775	7.797 ± 0.538				7.299 ± 0.503	34.080 ± 2.350				21.228 ± 1.464		78.711 ± 0.433	0.301 ± 0.001
114	*Verbena × hybrid*							25.007 ± 1.137	48.224 ± 2.200	6.914 ± 0.426			22.666 ± 1.051		102.689 ± 0.424	0.297 ± 0.005
115	*V. agnus- castus*								4.909 ± 0.103				2.582 ± 0.153		7.491 ± 0.092	0.038 ± 0.001
Blue flowers																
116	*A. africanus*					3.648 ± 0.460			4.478 ± 0.373						8.125 ± 0.069	
117	*C. althaeoides*														nd	
118	*S. ionantha*								1.911 ± 0.145						1.911 ± 0.145	
119	*S. aemula*			18.220 ± 1.227		3.139 ± 0.152		4.112 ± 0.277	16.117 ± 1.085						41.588 ± 0.228	
120	*A. foeniculum*								47.885 ± 3.833				35.877 ± 2.872		83.763 ± 0.516	0.607 ± 0.003
121	*L. angustifolia*	S	17.478 ± 0.822						19.190 ± 0.893		4.540 ± 0.373	19.347 ± 0.224	59.506 ± 1.492	12.258 ± 0.841	132.320± 0.513	0.722 ± 0.001
122	*R. officinalis*	S							14.888 ± 1.021						14.888± 1.021	
123	*Passiflora × belotti*					4.818 ± 0.454			7.122 ± 0.580				21.554 ± 2.032	15.638 ± 0.216	49.133± 0.436	0.196 ± 0.003
124	*P. vulgaris*								1.736 ± 0.412						1.736 ± 0.412	
125	*Petunia × hybrida*								23.061± 1.631				16.645 ± 0.021		39.707± 0.136	0.230 ± 0.000

S, saponified; nd, not detectable.

**Table 7:** Phenolic compound contents (mg/g dry weight) of white, yellow, and orange flowers.

	Species	Gallic	*p*-Hydroxybe.	*m*-Coumaric	*p*-Coumaric	Vanillic	Caffeic	Syringic	Chlorogenic	Ferulic	Naringin	Crisin	Quercitrin	Myricetin	Quercetin	Kaempferol	Total
White flowers																	
1	*S. montanum*																nd
2	*C. comosum*		7.868 ± 0.718	2.802 ± 0.053									4.061 ± 0.092		0.475 ± 0.039	4.249 ± 0.038	21.110 ± 1.393
3	*A. africanus*			8.648 ± 0.934									2.032 ± 0.081		1.444 ± 0.249	0.890 ± 0.022	13.013 ± 1.287
4	*C. sativum*	0.242 ± 0.023	0.560 ± 0.008	1.185 ± 0.042	0.372 ± 0.020								0.089 ± 0.001				2.448 ± 0.094
5	*N. oleander*	0.103 ± 0.003		2.999 ± 0.031					3.091 ± 0.052	0.443 ± 0.020	0.215 ± 0.014		3.428 ± 0.372		3.339 ± 0.029		13.618 ± 0.521
6	*T. jasminoides*		1.719 ± 0.014	0.398 ± 0.085			0.990 ± 0.001				0.323 ± 0.0036		0.802 ± 0.154				4.132 ± 0.028
7	*H. arborescens*	0.476 ± 0.013		0.456 ± 0.001					0.227 ± 0.020								1.158 ± 0.163
8	*M. incana*			5.710 ± 1.153													5.710 ± 1.153
9	*C. shetleri*			2.329 ± 0.079	0.218 ± 0.020								1.338 ± 0.107		0.248 ± 0.004		4.133 ± 0.092
10	*D. chinensis*												5.655 ± 0.601		3.870 ± 0.253		9.525 ± 0.016
11	*G. paniculata*			4.277 ± 0.467									17.930 ± 2.552				22.208 ± 0.132
12	*C.s scammonia*		0.844 ± 0.053	1.611 ± 0.274					0.146 ± 0.004				1.472 ± 0.242		0.438 ± 0.090	4.884 ± 0.053	9.598 ± 0.073
13	*G. communis*				0.688 ± 0.085		0.123 ± 0.009		0.577 ± 0.010	0.587 ± 0.009							2.390 ± 0.129
14	*M. suaveolens*		0.601 ± 0.035										1.337 ± 0.028		1.247 ± 0.013		3.863 ± 0.704
15	*M. grandiflora*		0.168 ± 0.001	0.575 ± 0.035													0.744 ± 0.036
16	*J. sambac*			5.913 ± 0.217	0.290 ± 0.003					0.146 ± 0.005			2.211 ± 0.040		0.261 ± 0.016		9.219 ± 0.028
17	*P. aphrodite*	1.063 ± 0.054			0.201 ± 0.010								6.565 ± 0.214				7.954 ± 0.886
18	*P. auriculata*		19.895 ± 2.118										17.592 ± 0.561		22.356 ± 0.618		59.843 ± 0.252
19	*F. ×* *ananassa*			0.472 ± 0.011									24.183 ± 0.625		16.983 ± 0.321		41.628 ± 0.127
20	*Rosa hybrid*	4.033 ± 0.199											1.302 ± 0.029		1.499 ± 0.351		12.501 ± 1.098
21	*C. annuum*		0.358 ± 0.068		0.737 ± 0.106		1.372 ± 0.069		1.162 ± 0.044	0.545 ± 0.007			4.978 ± 0.057		0.547 ± 0.010		9.699 ± 0.0877
22	*S. laxum*		0.213 ± 0.076	0.301 ± 0.001			0.099 ± 0.001		0.953 ± 0.052								1.266 ± 0.132
23	*A. citriodora*		1.944 ± 0.036				2.869 ± 0.028		3.748 ± 0.068				4.401 ± 0048				15.643 ± 0.001
24	*L. camara*			7.556 ± 0.026								3.402 ± 0.020			2.170 ± 0.115	5.922 ± 0.173	19.051 ± 0.033
Yellow flowers																	
25	*A. commutatum*					0.084 ± 0.022			0.220 ± 0.009				0.121 ± 0.034		0.162 ± 0.032		0.588 ± 0.097
26	*A. tinctoria*			2.345 ± 0.013					6.767 ± 0.228	0.495 ± 0.041			1.309 ± 0.013		0.152 ± 0.004		11.066 ± 0.615
27	*D. coccinea*		6.902 ± 0.027	4.738 ± 0.227									1.325 ± 0.303		0.721 ± 0.078	3.570 ± 0.2488	15.733 ± 0.0774
28	*D. pinnata*	0.340 ± 0.022		1.402 ± 0.081					1.325 ± 0.130	0.721 ± 0.078			0.597 ± 0.037		0.824 ± 0.139	4.475 ± 0.542	9.685 ± 0.012
29	*D. tenuifolia*												2.186 ± 0.006		3.075 ± 0.438	2.439 ± 0.388	7.701 ± 0.083
30	*C. sativa*		0.239 ± 0.014										1.719 ± 0.130		0.233 ± 0.010		2.192 ± 0.155
31	*E. japonicus*	0.245 ± 0.090		0.190 ± 0.001								0.225 ± 0.031	0.649 ± 0.010		0.251 ± 0.006		1.858 ± 0.0218
32	*S. japonica*		0.813 ± 0.039	0.179 ± 0.007	0.409 ± 0.018			0.427 ± 0.006					3.395 ± 0.146		0.972 ± 0.035	2.669 ± 0.347	9.167 ± 0.742
33	*S. papillosa*																nd
34	*P. stenoptera*	12.605 ± 1.193						1.220 ± 0.019	1.529 ± 0.134				0.505 ± 0.068		0.131 ± 0.027		18.078 ± 0.119
35	*O. basilicum*						0.207 ± 0.013								0.328 ± 0.032		0.640 ± 0.0050
36	*G. arboreum*				0.4360 ± 0.025					0.308 ± 0.002			5.621 ± 0.766		1.507 ± 0.029		7.872 ± 0.011
37	*P. major*						0.751 ± 0.051		0.856 ± 0.058				21.226 ± 1.503				22.833 ± 0.161
38	*F. aubertii*	0.806 ± 0.028						0.091 ± 0.006					0.191 ± 0.024	0.399 ± 0.018	0.136 ± 0.018		1.725 ± 0.089
39	*P. oleracea*		1.302 ± 0.101	2.425 ± 0.001	0.125 ± 0.007				0.457 ± 0.049								4.276 ± 0.053
40	*G. jasminoides*									0.600 ± 0.026	0.316 ± 0.001	0.407 ± 0.023			0.311 ± 0.004		1.956 ± 0.025
41	*S. lycopersicum*		1.849 ± 0.297		0.435 ± 0.009								0.620 ± 0.072		0.691 ± 0.059		3.395 ± 0.436
42	*L. camara*		1.034 ± 0.107	2.665 ± 0.043			1.175 ± 0.122						2.412 ± 0.003		2.126 ± 0.040	1.634 ± 0.011	10.862 ± 0.072
Orange flowers																	
43	*J. aurea*																nd
44	*T. capensis*		0.350 ± 0.070										0.773 ± 0.117		0.208 ± 0.005		1.331 ± 0.019
45	*D.a affinis*																nd
46	*D. brochidodroma*																nd
47	*P. granatum*			9.103 ± 0.533	10.080 ± 0.358				8.421 ± 0.159				56.464 ± 2.298		22.133 ± 1.821		146.937 ± 0.669
48	*R. alpinia*																nd

nd, not detectable.

**Table 8:** Phenolic compound contents (mg/g dry weight) of red and pink flowers.

	Species	Gallic	*p*-Hydroxybe.	*m*-Coumaric	*p*-Coumaric	Vanillic	Caffeic	Syringic	Chlorogenic	Ferulic	Naringin	Crisin	Quercitrin	Myricetin	Quercetin	Kaempferol	Total
Red flowers																	
49	*A. squarrosa*																nd
50	*C. argentea*	0.654 ± 0.026	1.660 ± 0.007	0.665 ± 0.007		0.079 ± 0.004	0.098 ± 0.013		0.179 ± 0.002				3.558 ± 0.286		0.251 ± 0.008	0.572 ± 0.018	7.715 ± 0.044
51	*C.s roseus*			1.190 ± 0.560	16.458 ± 1.017		0.691 ± 0.069						0.953 ± 0.004		0.791 ± 0.056	6.029 ± 0.254	26.476 ± 2.014
52	*N. oleander*			4.114 ± 0.118					6.622 ± 0.307	0.764 ± 0.057			1.347 ± 0.013		4.388 ± 0.041	4546 ± 0.385	21.781 ± 1.893
53	*A. andraeanum*			0.361 ±0.051									6.192 ± 0.177				7.664 ± 0.258
54	*I. balsamina*												1.503 ± 0.285		0.133 ± 0.028	1.394 ± 0.190	3.244 ±0.052
55	*I. walleriana*			3.120 ± 0.145									1.195 ± 0.145			3.611 ± 0.004	8.428 ± 0.384
56	*B. cavaleriei*	1.311 ± 0.060		0.265 ± 0.013	0.641 ± 0.030								1.417 ± 0.071				4.330 ± 0.213
57	*B. andraeanum*	0.360 ± 0.046	0.273 ± 0.019	0.414 ± 0.048	0.207 ± 0.001								1.984 ± 0.013		2.217 ± 0.099	4.191 ± 0.049	9.646 ± 0.274
58	*Begonia × tuberhybrida*												1.650 ± 0.242	3.306 ± 0.069	0.642 ± 0.039	2.730 ± 0.205	8.693 ± 0.874
59	*D. caryophyllus*			13.167 ± 0.241		1.512 ± 0.095							0.331 ± 0.031		0.396 ± 0.071		15.405 ± 2.662
60	*R. simsii*				0.279 ± 0.057				0.138 ± 0.013				0.361 ± 0.003		0.131 ± 0.003		1.142 ± 0.010
61	*E. rubra*			0.042 ± 0.002									0.238 ± 0.068		0.326 ± 0.050		0.600 ± 0.122
62	*E. milii*	4.882 ± 0.502		0.347 ± 0.018									0.724 ± 0.035		0.167 ± 0.018		7.414 ± 0.062
63	*B. macrophylla*																nd
64	*P. peltatum*	14.741 ± 0.122		2.216 ± 0.011	1.103 ± 0.102		0.248 ± 0.001		0.440 ± 0.012		8.415 ± 0.012						32.452 ± 0449
65	*Pelargonium × hortorum*	7.142 ± 2.789		2.011 ± 0.003	1.406 ± 0.147		1.371 ± 0.185				18.655 ± 0.296			3.081 ± 0.032			68.975 ± 4.079
66	*S. splendens*		1.182 ± 0.014	0.683 ± 0.008			4.311 ± 0.053		0.213 ± 0.003	0.801 ± 0.100							7.245 ± 0.078
67	*M. arboreus*			3.440 ± 0.450									3.429 ± 0.334	0.510 ± 0.071		6.166 ± 0.412	14.824 ± 1.379
68	*F. magellanica*	4.327 ± 0.154		11.080 ± 0.105							1.555 ± 0.022		23.538 ± 0.242				42.488 ± 1.335
69	*Rosa hybrid*	3.194 ± 0.642		4.989 ± 0.065							0.793 ± 0.015		0.970 ± 0.075		1.065 ± 0.248	6.339 ± 0.029	19.201 ± 1.513
70	*P. rhoeas*																nd
71	*W. coccinea*																nd
72	*A. majus*		0.911 ± 0.002	2.443 ± 0.312											0.146 ± 0.019	4.354 ± 0.197	8.962 ± 0.106
73	*R. equisetiformis*			2.577 ± 0.193											0.137 ± 0.032		4.795 ± 0.061
74	*Petunia × hybrid*			11.005 ± 0.795					2.724 ± 0.123								13.729 ± 0.191
75	*L. camara*			9.204 ± 0.120			0.911 ± 0.003	1.001 ± 0.021				0.603 ± 0.003	5.833 ± 0.804		2.306 ± 0.011	2.642 ± 0.333	22.478 ± 0.301
76	*Verbena × hybrid*			2.493 ± 0.176	1.373 ± 0.001				0.949 ± 0.139			1.187 ± 0.081	9.888 ± 0.148		4.310 ± 0.214		21.283 ± 0.834
Pink flowers																	
77	*C. argentea*	1.635 ± 0.068	6.172 ± 0.254		0.239 ± 0.022								0.218 ± 0.001		0.634 ± 0.013	4.615 ± 0.074	13.765 ± 0.437
78	*N. oleander*			5.514 ± 0.513		0.384 ± 0.086			7.902 ± 0.090	1.123 ± 0.101			0.464 ± 0.069		0.290 ± 0.045		15.677 ± 0.904
79	*B. argentea*												0.214 ± 0.021		0.274 ± 0.029		0.488 ± 0.051
80	*G. hybrid*						0.491 ± 0.065	0.473 ± 0.003				0.118 ± 0.019					1.229 ± 0.097
81	*D. caryophyllus*			8.479 ± 0.385									0.714 ± 0.032	1.527 ± 0.069	0.087 ± 0.004		10.807 ± 0.049
82	*S. officinalis*		1.733 ± 0.160										8.728 ± 0.943				10.461 ± 1.103
83	*R. simsii*				0.279 ± 0.057				0.138 ± 0.013				0.361 ± 0.003		0.131 ± 0.003		1.142 ± 0.102
84	*T. cernuum*			0.279 ± 0.005	0.166 ± 0.004								1.354 ± 0.079		0.605 ± 0.031		2.674 ± 0.187
85	*E. grandiflorum*		0.257 ± 0.008	0.338 ± 0.086									1.713 ± 0.141				3.105 ± 0.271
86	*P. domesticum*	15.733 ± 0.120		6.495 ± 0.152	0.310 ± 0.011			1.345 ± 0.086			3.485 ± 0.484						32.678 ± 2.198
87	*Pelargonium × hortorum*	22.520 ± 1.728		3.162 ± 0.070	3.704 ± 0.073						19.506 ± 1.375			6.291 ± 2.100			55.183 ± 5.346
88	*H. petiolaris*			10.889 ± 0.114			34.229 ± 2.096		6.566 ± 0.175		3.183 ± 0.162		4.350 ± 0.224		2.154 ± 0.115		61.371 ± 4.260
89	*C. hyssopifolia*											11.312 ± 0.867					11.312 ± 0.867
90	*L. indica*			0.424 ± 0.006				2.667 ± 0.395									3.091 ± 0.484
91	*G. arboreum*	0.367 ± 0.010	2.762 ± 0.326						0.545 ± 0.036				0.862 ± 0.073		0.741 ± 0.034		5.276 ± 0.479
92	*M. jalapa*		4.530 ± 0.048	2.230 ± 0.022					0.729 ± 0.044	0.369 ± 0.012			0.332 ± 0.013		0.373 ± 0.009	0.922 ± 0.123	9.485 ± 0.271
93	*P. aphrodite*			10.146 ± 0.349	1.614 ± 0.049								17.202 ± 0.282		3.136 ± 0.020		39.969 ± 1.073
94	*P. oleracea*		0.402 ± 0.111	4.008 ± 0.160	0.125 ± 0.007				0.347 ± 0.050								4.389 ± 0.021
95	*Rosa hybrid*			5.799 ± 0.424			0.333 ± 0.014						0.351 ± 0.050				7.770 ± 0.033
96	*Verbena ×hybrid*			18.240 ± 1.965								1.470 ± 0.169	6.890 ± 0.668		0.198 ± 0.014		26.797 ± 2.816

nd, not detectable.

**Table 9:** Phenolic compound contents (mg/g dry weight) of lilac and blue flowers.

	Species	Gallic	*p*-Hydroxybe.	*m*-Coumaric	*p*-Coumaric	Vanillic	Caffeic	Syringic	Chlorogenic	Ferulic	Naringin	Crisin	Quercitrin	Myricetin	Quercetin	Kaempferol	Total
Lilac flowers																	
97	*A. schoenoprasum*		2.156 ± 0.0244	2.736 ± 0.274	0.190 ± 0.007				0.031 ± 0.006				0.894 ± 0.003		1.284 ± 0.010	1.978 ± 0.125	9.269 ± 0.670
98	*C. roseus*		1.680 ± 0.094	7.023 ± 0.685					2.263 ± 0.169				1.021 ± 0.058		2.525 ± 0.150	14.559 ± 1.223	29.137 ± 3.556
99	*C. seridis*		0.987 ± 0.138	0.544 ± 0.030			0.532 ± 0.024		1.087 ± 0.001				7.465 ± 0.174	0.288 ± 0.019	2.044 ± 0.136	3.055 ± 0.612	16.001 ± 2.364
100	*C. intybus*		1.433 ± 0.123	3.938 ± 0.175	0.388 ± 0.069		0.512 ± 0.004		0.911 ± 0.061	0.446 ± 0.014	0.415 ± 0.053						9.891 ± 0.848
101	*O. fruticosum*			0.898 ± 0.182									0.658 ± 0.071		0.561 ± 0.094	0.729 ± 0.133	3.051 ± 0.501
102	*A. montanum*			0.105 ± 0.024					0.871 ± 0.098		0.191 ± 0.023						1.677 ± 0.253
103	*C. carpatica*			4.031 ± 0.349									0.621 ± 0.008				4.785 ± 0.501
104	*P. domesticum*	17.440 ± 1.088		10.666 ± 0.513				2.397 ± 0.088			2.434 ± 0.121		0.711 ± 0.059	1.045 ± 0.116	1.297 ± 0.015		37.965 ± 0.125
105	*P.* *×* *hortorum*	27.709 ± 0.061		6.280 ± 0.437	1.537 ± 0.219								1.672 ± 0.002		2.599 ± 0.125		39.797 ± 0.096
106	*M.* *× piperita*				0.062 ± 0.003				0.083 ± 0.004		0.210 ± 0.013				0.133 ± 0.007		0.488 ± 0.027
107	*O. basilicum*							0.197 ± 0.008									0.197 ± 0.008
108	*H. syriacus*			10.489 ± 0.107									19.139 ± 1.870		0.318 ± 0.036		29.946 ± 2.013
109	*B. spectabili*		3.844 ± 0.218								0.352 ± 0.015		7.161 ± 0.409		4.422 ± 0.269		15.779 ± 1.334
110	*L. sinuatum*	0.393 ± 0.077											0.350 ± 0.008		0.120 ± 0.030	0.972 ± 0.073	2.036 ± 0.171
111	*F. aubertii*	0.749 ± 0.011						0.160 ± 0.010					0.377 ± 0.022	0.711 ± 0.092	0.388 ± 0.130		2.659 ± 0.276
112	*Petunia* *×* *hybrida*		0.197 ± 0.010		0.683 ± 0.082	0.187 ± 0.020				0.846 ± 0.052			0.647 ± 0.020		1.322 ± 0.102	0.909 ± 0.035	4.791 ± 0.320
113	*S. rantonnetti*		1.213 ± 0.083						0.612 ± 0.017	0.685 ± 0.072			0.391 ± 0.002		0.278 ± 0.002		3.180 ± 0.177
114	*Verbena* *×* *hybrid*			6.323 ± 0.143	0.504 ± 0.029		1.467 ± 0.056		0.925 ± 0.022			1.282 ± 0.158	3.519 ± 0.355		8.950 ± 0.526		24.044 ± 2.346
115	*V.agnus- castus*		5.337 ± 0.337	15.534 ± 0.790		0.564 ± 0.010						5.344 ± 0.168	1.580 ± 0.035		0.363 ± 0.030		28.994 ± 2.552
Blue flowers																	
116	*A. africanus*			6.751 ± 0.339									1.452 ± 0.069		1.032 ± 0.089	3.663 ± 0.171	13.687 ± 0.736
117	*C. althaeoides*			0.029 ± 0.004			3.938 ± 0.309		0986 ± 0.127	2.995 ± 0.387						1.568 ± 0.203	9.516 ± 1.231
118	*S. ionantha*										1.519 ± 0.037	19.628 ± 2.488	2.940 ± 0.062		1.641 ± 0.200	10.830 ± 0.428	37.201 ± 3.850
119	*S. aemula*		2.659 ± 0.128	0.534 ± 0.010					0.529 ± 0.078			0.851 ± 0.064	1.322 ± 0.048		0.837 ± 0.171		7.257 ± 0.525
120	*A. foeniculum*			3.469 ± 0.258							0.648 ± 0.013						6.628 ± 0.470
121	*L. angustifolia*			1.614 ± 0.235			1.666 ± 0.231						1.207 ± 0.067		0.413 ± 0.029		5.634 ± 0.834
122	*R. officinalis*			1.634 ± 0.050							4.981 ± 0.490		1.035 ± 0.020				7.651 ± 0.560
123	*P.* *× belotti*			6.005 ± 0.614									0.359 ± 0.024				6.364 ± 1.475
124	*P. vulgaris*			4.490 ± 0.097													4.490 ± 0.097
125	*Petunia* *×* *hybrida*								2.300 ± 0.251	0.578 ± 0.013			0.554 ± 0.050		2.966 ± 0.005	0.948 ± 0.041	7.850 ± 0.048

## Data Availability

The datasets generated for this study are available on request to the corresponding author.
